# *MOXD1* is a lineage-specific gene and a tumor suppressor in neuroblastoma

**DOI:** 10.1126/sciadv.ado1583

**Published:** 2024-06-21

**Authors:** Elina Fredlund, Stina Andersson, Elien Hilgert, Ezequiel Monferrer, Guadalupe Álvarez-Hernán, Sinan Karakaya, Siebe Loontiens, Jan Willem Bek, Tomas Gregor, Estelle Lecomte, Emma Magnusson, Enrika Miltenyte, Marie Cabirol, Michail Kyknas, Niklas Engström, Marie Arsenian Henriksson, Emma Hammarlund, Jared S. Rosenblum, Rosa Noguera, Frank Speleman, Johan van Nes, Sofie Mohlin

**Affiliations:** ^1^ Division of Pediatrics, Department of Clinical Sciences, Lund University, Lund, Sweden.; ^2^ Lund University Cancer Center, Lund University, Lund, Sweden.; ^3^ Lund Stem Cell Center, Lund University, Lund, Sweden.; ^4^ Center for Medical Genetics, Ghent University, Ghent, Belgium.; ^5^ Department of Pathology, Medical School, University of Valencia-INCLIVA Biomedical Health Research Institute, Valencia, Spain.; ^6^ Low Prevalence Tumors, Centro de Investigación Biomédica En Red de Cáncer (CIBERONC), Instituto de Salud Carlos III, Madrid, Spain.; ^7^ Department of Microbiology, Tumor and Cell Biology (MTC), Biomedicum B7, Karolinska Institute, Stockholm, Sweden.; ^8^ Department of Laboratory Medicine, Lund University, Lund, Sweden.; ^9^ Section on Medical Neuroendocrinology, Eunice Kennedy Shriver National Institute of Child Health and Development, National Institutes of Health, Bethesda, MD 20892, USA.; ^10^ Department of Oncogenomics, Academic Medical Center, University of Amsterdam, Amsterdam, Netherlands.

## Abstract

Neuroblastoma is a childhood developmental cancer; however, its embryonic origins remain poorly understood. Moreover, in-depth studies of early tumor-driving events are limited because of the lack of appropriate models. Herein, we analyzed RNA sequencing data obtained from human neuroblastoma samples and found that loss of expression of trunk neural crest–enriched gene *MOXD1* associates with advanced disease and worse outcome. Further, by using single-cell RNA sequencing data of human neuroblastoma cells and fetal adrenal glands and creating in vivo models of zebrafish, chick, and mouse, we show that MOXD1 is a determinate of tumor development. In addition, we found that *MOXD1* expression is highly conserved and restricted to mesenchymal neuroblastoma cells and Schwann cell precursors during healthy development. Our findings identify *MOXD1* as a lineage-restricted tumor-suppressor gene in neuroblastoma, potentiating further stratification of these tumors and development of novel therapeutic interventions.

## Teaser

*MOXD1* is a tumor-suppressor gene and a lineage-specific marker for immature and mesenchymal cells in neuroblastoma.

## INTRODUCTION

Neuroblastoma is the most common cause of pediatric cancer–related death, with most patients diagnosed before 5 years of age (*1*). These tumors are typically found in the trunk, particularly the adrenal gland or paraspinal sympathetic ganglia (*2*–*4*). Despite recent clinical trials with improved treatment protocols, 3-year event-free survival rates for high-risk neuroblastoma remains around 50 to 60% (*5*, *6*). The location and age of onset of neuroblastoma reflects its embryonic origin from neural crest progenitor cells. Patients with neuroblastoma are categorized on the basis of a composite of prognostic factors aimed at addressing risk and treatment approaches. Among the prognostic factors are age at diagnosis, where older (over 18 months) patients present with worse outcome, status of the *MYCN* oncogene, and stage of disease (*7*). Furthermore, the high-risk neuroblastoma patients with a chromosomal loss of distal 6q demonstrate a notably diminished survival probability (*8*). A subset of high-risk neuroblastomas (denoted as stage 4s) with widespread metastasis at diagnosis may undergo spontaneous and complete regression without therapeutic intervention. While neuroblastomas are highly malignant, other neuroblastic tumors such as ganglioneuroblastomas and ganglioneuromas, which similarly consist of abnormally growing and migrating immature neuroblasts, manifest as benign tumors and frequently remain asymptomatic.

During normal development, the neural crest arises from the posterior of the neural tube, which develops into the brain and spinal cord, and undergo epithelial to mesenchymal transition (EMT). This results in neural crest migration away from the neural tube and into the rest of the body to form specialized cells throughout other organ systems, including the chromaffin cells of the adrenal medulla, peripheral sympathetic nerves, and Schwann cells, which support the peripheral nerves. Early pluripotent neural crest progenitors develop into four groups of multipotent neural crest cells with unique fates—cranial, vagal, trunk, and sacral neural crest (*9*, *10*). Neuroblastoma, primarily found in the trunk, is believed to develop from trunk neural crest; however, the founder cell types of neuroblastomas are still unknown. To fully understand the initiation and early progression of neuroblastoma, we must identify genetic and epigenetic signatures and study the impact of introducing these defects on altering normal neural crest development.

Recent studies of the epigenomic and transcriptomic profiles of neuroblastoma identified two distinct cellular phenotypes that are suggested to affect neuroblastoma development and progression: lineage-committed adrenergic (ADRN) and immature neural crest–like mesenchymal (MES) phenotypes (*11*, *12*). ADRN cells have been found to grow more aggressively in mouse models, whereas MES cells grew more slowly and demonstrated increased resistance to treatment with chemotherapy and ALK inhibitors (*12*–*14*). Further, established neuroblastoma cell lines can be categorized as MES, ADRN, or mixed/intermediate on the basis of their expression profiles (*12*, *15*, *16*). While the characterization of these phenotypes represents a critical step toward understanding neuroblastoma development, their role in tumor evolution remains unclear as cells may switch between the two phenotypes. Other studies have recently attempted to map the cellular composition of normal developing human and murine adrenal medulla to the features of neuroblastoma cells with single-cell RNA sequencing (scRNA-seq) and have found that neuroblastoma cells relate to various stages of normal sympathetic neuron maturation (*15*, *17*–*28*). However, discrepancies in data interpretation, usage of different species and developmental time points, and analyses restricted to neuroblastoma subtypes warrant further investigation. Thus far, the classification of neuroblastoma as defined by their cellular origin is lacking.

Two studies of neural crest in avian development using in situ hybridization and bulk RNA-seq showed that *Monooxygenase DBH-like 1* (*MOXD1*), located at chromosome 6q23.2, is expressed in migrating trunk neural crest cells (*29*, *30*). While the essential functions performed by dopamine beta-hydroxylate (DBH), which belongs to the same copper monooxygenase family, in chromaffin cells and neurons of the central nervous system are well known, the function of MOXD1 remains elusive. *MOXD1* is, however, highly conserved between humans and multiple translational models including chickens, mice, and zebrafish. Thus, herein, we used these translational models to evaluate the impact of altered *MOXD1* expression on neuroblastoma formation together with combined data obtained from correlative single-cell studies and patient tumor material.

## RESULTS

### The correlation between low *MOXD1* expression and worse patient prognosis is a neuroblastoma-specific feature

**Fig. 1.**
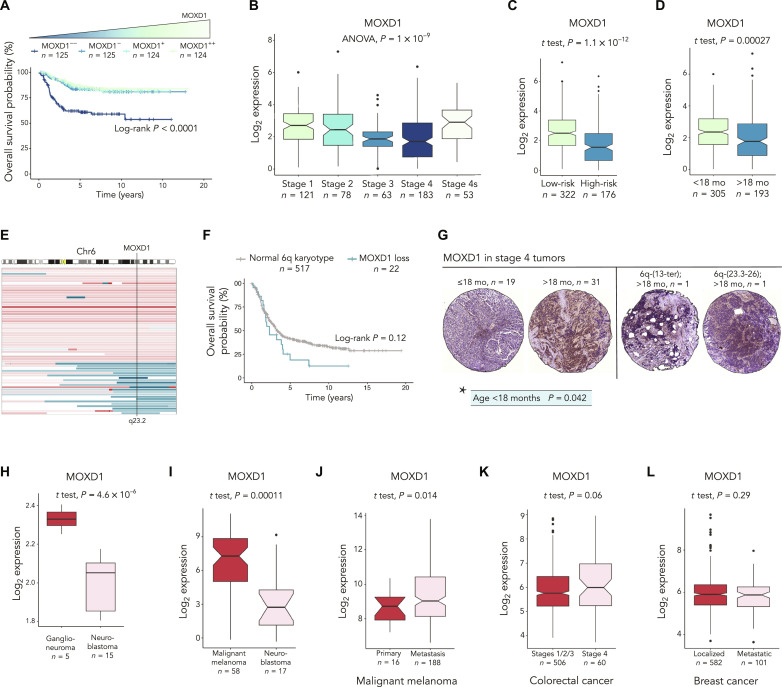
The correlation between low *MOXD1* expression and worse patient prognosis is a neuroblastoma specific feature. (**A**) Kaplan-Meier survival curves with log-rank *P* value for patients with neuroblastoma in the SEQC cohort (*n* = 498) stratified into quartiles based on the *MOXD1* mRNA expression levels. (**B** and **C**) *MOXD1* expression in patients with neuroblastoma (SEQC cohort) stratified according to the INSS stages (B) or low-risk or high-risk (C). (**D**) *MOXD1* expression in patients with neuroblastoma (SEQC cohort) stratified according to the age at diagnosis (<18 months versus >18 months). (**E**) Detailed view of gains (red) and losses (blue) on chromosome 6 from high-risk patients in the Depuydt cohort (*n* = 556). The black line indicates the chromosomal location of *MOXD1* (6q23.2). Maximum size of aberration was set to 180 Mb. (**F**) Kaplan-Meier plot of overall survival of patients with high-risk neuroblastoma (Depuydt; *n* = 539). Patients stratified by loss of *MOXD1* or no loss of *MOXD1*. Patients with distal 6q mutations not affecting *MOXD1* and patients lacking overall survival information were excluded. (**G**) Representative images of tumor cores stained for MOXD1 in different age groups [≤ 18 months versus > 18 months at diagnosis (left)] and tumors with loss of 6q (right). *Statistical analysis of significant correlations between *MOXD1* expression and age at diagnosis. (**H**) *MOXD1* expression in patients with ganglioneuroma versus neuroblastoma. (**I**) Expression of *MOXD1* in malignant melanoma versus neuroblastoma; data extracted from the Cancer Cell Line Encyclopedia. (**J**) Expression of *MOXD1* in the primary or metastatic malignant melanoma. (**K**) Expression of *MOXD1* in colorectal cancer of stage 4 versus stages 1/2/3. (**L**) Expression of *MOXD1* in localized versus metastatic breast cancer. (H) to (L) Number of patients (*n*) is depicted in the graphs.

We investigated the relationship between prognosis and the transcriptomic profile in three independent publicly available cohorts of patients with neuroblastoma (Fig. 1 and fig. S1) and found that expression of *MOXD1* is an independent predictor of survival in these patients (fig. S1, A to D). Stratifying patients from the Sequencing Quality Control (SEQC) cohort, previously reported by Zhang *et al.* (*31*), encompassing 498 individuals with neuroblastoma based on their *MOXD1* expression revealed that tumors with the lowest (first quartile) *MOXD1* expression had the worse overall survival (Fig. 1A). This relationship was confirmed in two additional neuroblastoma patient cohorts (fig. S1, A and B) (*32*, *33*). Further, we found that, in all three cohorts, low expression of *MOXD1* predicted worse event-free survival (fig. S1, E to G). We further stratified the *MOXD1* expression in the tumors from these individuals according to the International Neuroblastoma Staging System (INSS) stages and low- and high-risk groups and observed a negative correlation between *MOXD1* and more advanced tumor stage and high-risk neuroblastomas (Fig. 1, B and C, and fig. S1, H and I). We observed that expression of *MOXD1* increased in individuals with stage 4s disease, which presents with widespread metastatic disease at diagnosis but that remarkably regresses spontaneously without treatment (Fig. 1B and fig. S1, H and I). Moreover, we detected that the *MOXD1* expression was decreased in individuals older than 18 months (Fig. 1D and fig. S1, J and K), an age category that is associated with worse prognosis. Stratifying patients according to their *MYCN* status, we observed that the *MOXD1* expression was lower in *MYCN*-amplified tumors (fig. S1, L to N). Together, the results show that low expression of the trunk neural crest–enriched gene *MOXD1* is an independent prognostic marker of poor outcome in neuroblastoma.

Next, we evaluated a subset of individuals with neuroblastoma for possible causes of low *MOXD1* expression and found altered copy number profile, suggesting the presence of chromosomal losses surrounding the *MOXD1* locus (6q23.2, fig. S2A). Furthermore, in a cohort previously presented by Depuydt *et al.* (*8*) containing information on distal 6q chromosomal loss, we found that a subset of high-risk patients had a loss of *MOXD1* of which correlated with an extremely low survival probability (Fig. 1, E and F). We confirmed that *MOXD1* expression (as part of the gene set located at 6q23.2) was lower in tumor cells with loss of distal 6q (fig. S2B). Together, this demonstrates that the low expression or loss of *MOXD1* is correlated with unfavorable disease in neuroblastoma.

### MOXD1 protein expression is correlated with age in stage 4 tumors

We stained a tissue microarray (TMA) consisting of tumor tissue from a cohort of 50 patients with stage 4 and *MYCN*-amplified neuroblastomas by immunohistochemistry (Fig. 1G). Two tumors carried a loss of the same locus of 6q as noted above (Fig. 1E). Albeit this cohort contains few tumors with loss of 6q, the numbers (2 of 50; 4%) are consistent with a previously published cohort (5.9%) (*8*). We observed a heterogenous expression pattern of MOXD1 across all tumors. Notably, the tumors bearing increased MOXD1 expression demonstrated increased variation in intratumor expression intensity. Although there was no correlation to the outcome within this patient group with an already poor prognosis, the expression of the MOXD1 protein correlated to the age at diagnosis, with lower heterogeneity of MOXD1 observed in children aged below 18 months (Fig. 1G). Both cases with loss of 6q were older than 18 months, consistent with the findings observed in patients presenting with poor prognosis. Tumors with loss of 6q presented with ~30% positive cells each, but only 1 and 4%, respectively, of them were stained with high intensity. These tumor samples were, thus, not depleted of MOXD1, indeed highlighting intratumor heterogeneity and the presence of surrounding nontumor cells in these tissues.

### The prognostic role of *MOXD1* is tumor-type specific

We evaluated whether low *MOXD1* expression is specific to neuroblastomas by analyzing data from multiple other neural crest–derived and nonneural crest–derived cancers. We found that *MOXD1* expression was significantly lower in neuroblastomas than in ganglioneuromas [Fig. 1H; sequencing data previously presented by Tao *et al.* (*34*)]. Malignant melanomas also derive from trunk neural crest, however, from cells migrating dorsally, as compared to neuroblastomas that descend from ventrally migrating cells. Comparing neuroblastoma to malignant melanoma using data from the Cancer Cell Line Encyclopedia cohort (*35*) showed that the *MOXD1* expression was significantly lower in neuroblastoma (Fig. 1I). We noted that there was a small increase in expression of *MOXD1* in metastatic melanoma compared to primary lesions (Fig. 1J) using a dataset previously presented by Cabrita *et al.* (*36*). We found no significant differences in *MOXD1* expression between stages 1/2/3 and stage 4, and localized and metastatic, in colorectal [data previously presented by Marisa *et al.* (*37*)] and breast [data previously presented by Cheng *et al.* (*38*)] cancer, respectively, which are both cancers believed to arise from nonneural crest lineages (Fig. 1, K and L).

### * MOXD1* is restricted to undifferentiated MES type cells in neuroblastoma

**Fig. 2.**
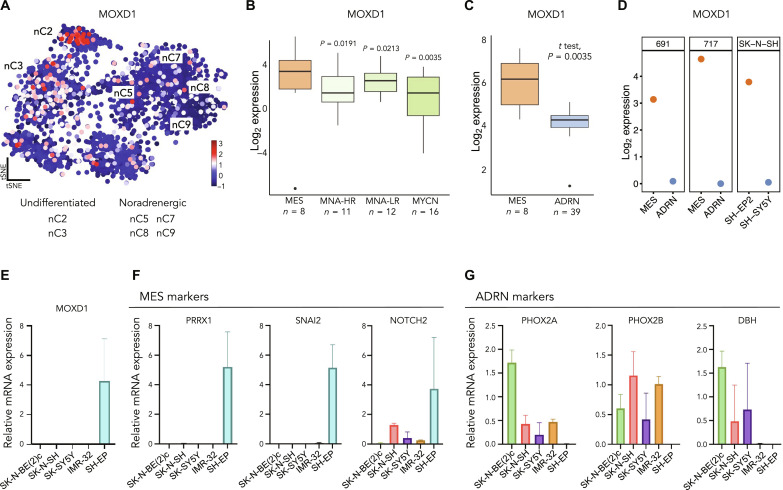
*MOXD1* is expressed in MES-like neuroblastoma cells. (**A**) Mapping of *MOXD1* mRNA expression in the t-distributed stochastic neighbor embedding (tSNE) of human neuroblastoma single nuclei data by Bedoya-Reina *et al.* (*17*). nCx (2, 3, 5, 7, 8, and 9) are selected clusters from the abovementioned published data. (**B**) Expression of *MOXD1* in the cohort from Gartlgruber *et al.* (*15*). The groups include MES (mesenchymal), NMNA-HR/LR (*MYCN* nonamplified high-risk/low-risk), and MYCN (*MYCN* amplified). Number of patients (*n*) is depicted in the graph, and *P* values were generated by analysis of variance (ANOVA), followed by Fisher’s least significant difference (LSD) test. (**C**) The *MOXD1* mRNA expression in neuroblastoma cells with ADRN and MES gene signatures. The number of samples (*n*) for each cell phenotype is depicted in the graph, and *P* value by *t* test as indicated. (**D**) RNA-seq–derived expression of *MOXD1* in the isogenic neuroblastoma cell line pairs. (**E**) *MOXD1* mRNA expression in three neuroblastoma cell lines with an ADRN phenotype (SK-N-BE(2)c, SH-SY5Y, and IMR-32), one with a mixed phenotype (SK-N-SH), and one with the MES phenotype (SH-EP) as assessed by qPCR. Error bars denote SD from *n* = 2 to 3 biologically independent repeats per cell line. (**F** and **G**) Expression of MES- (F) and ADRN- (G) associated genes as assessed by qPCR. *n* = 3 biologically independent repeats.

We analyzed a publicly available dataset of single-nuclei RNA-seq (snRNA-seq) from 11 neuroblastomas previously presented by Bedoya-Reina *et al.* (*17*) and found that expression of *MOXD1* was low in noradrenergic cell clusters and high in undifferentiated MES-like tumor cell clusters (Fig. 2A). Further, we analyzed another dataset containing chromatin immunoprecipitation sequencing data previously presented by Gartlgruber *et al.* (*15*) of 47 resected neuroblastoma samples and found that *MOXD1* expression was elevated in the MES and EMT group with previously unknown clinical and biological features (Fig. 2B). Consistent with this, by analyzing a publicly available dataset previously presented by van Groningen *et al.* (*12*), we found that the MES phenotypic cells had higher *MOXD1* levels than the ADRN phenotypic cells (Fig. 2C). We next analyzed the sequencing data from three isogenic MES and ADRN cell line pairs and found that all three cell lines with a MES phenotype expressed *MOXD1*, whereas this expression was absent in the corresponding ADRN phenotypic cells (Fig. 2D).

Next, we detected that *MOXD1*, as well as the MES-associated *PRRX1*, *SNAI2*, and *NOTCH2* genes were virtually restricted to the MES-like SH-EP cells (Fig. 2, E and F) by analyzing extracted RNA from five different conventional neuroblastoma cell lines. The three ADRN-like cell lines, as defined by van Groningen *et al.* (*12*), SK-N-BE(2)c, SH-SY5Y, and IMR-32, and the more mixed ADRN/MES cell line, as defined by Boeva *et al.* (*11*) and Gautier *et al.* (*39*), SK-N-SH were virtually devoid of *MOXD1* and MES-associated markers, but they instead expressed the ADRN-associated genes *PHOX2A*, *PHOX2B*, and *DBH* (Fig. 2, E to G). Given that we observed a low expression of MES markers in the mixed phenotype SK-N-SH cells, we further analyzed the available scRNA-seq data for this cell line from Gartlgruber *et al.* (*15*). Although these cells indeed express the markers of both subtypes, the expression of ADRN markers is slightly more dominant (fig. S3, A to C). *MOXD1* coexpressed with the MES marker *PRRX1* (fig. S3, A and B). We confirmed the MOXD1-restricted expression in three additional MES phenotypic cell lines using published bulk RNA-seq data (fig. S4) (*11*, *40*).

### Knockout of *MOXD1* in neuroblastoma cells enhances tumor burden in an in vivo model

**Fig. 3.**
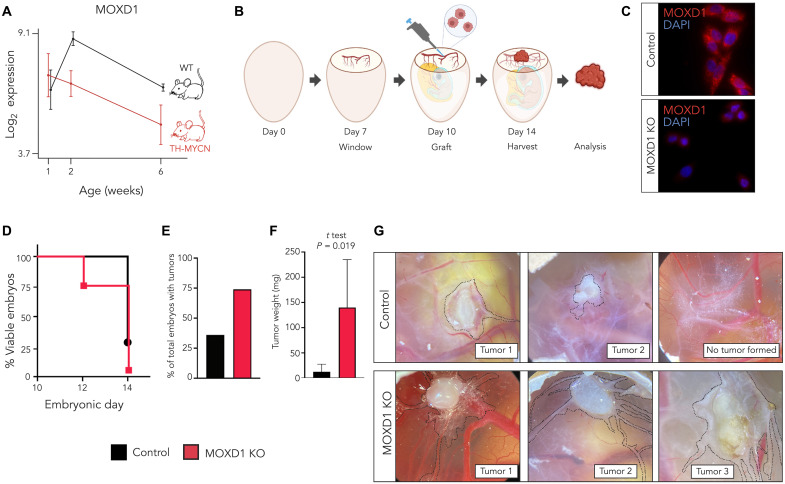
*MOXD1* knockout (KO) in neuroblastoma cells increases tumor formation in vivo. (**A**) *MOXD1* expression score analysis by RNA-seq [data from De Wyn *et al.* (2021)] of sympathetic ganglia from TH-MYCN mice (at 6 weeks of age, tumor tissue at the site of sympathetic ganglia was analyzed), and the corresponding sympathetic ganglia from wild type (WT) mice without tumors (*n* = 4 for each group and time point). The graph shows the mean expression score ± SD. (**B**) Schematic description of the CAM assay. Created with BioRender.com. (**C**) Confirmation of CRISPR-Cas9–mediated knockout of MOXD1 at protein level in neuroblastoma SH-EP cells by immunofluorescence. DAPI (4′,6-diamidino-2-phenylindole) was used to counterstain the nuclei. (**D**) Survival of chick embryos as presented by the percentage of viable embryos at the indicated time points. Eggs implanted with control (CTRL) cells are marked in black and MOXD1 KO in red. Implanted eggs: *n* = 28 CTRL and *n* = 38 MOXD1 KO. (**E**) Number of eggs with detectable tumors as presented by percentage at D14. There are no error bars due to the absolute numbers. Implanted eggs: *n* = 28 CTRL and *n* = 38 MOXD1 KO. (**F**) Weight in milligram (mg) of the dissected tumors. Error bars indicate SEM and *P* value determined by *t* test. Note that there are few eggs involved in this analysis due to the high number of dead embryos and spread tumors that could not be completely dissected. Weighed tumors at D14: *n* = 5 CTRL and *n* = 2 MOXD1 KO. (**G**) Representative images of the CAM in the respective group. Tumor area marked by dashed lines.

We analyzed microarray-derived expression data acquired from dissected sympathetic ganglia from hyperplastic lesions at week 2 and tumor tissue at week 6 from the *Tyrosine hydroxylase (TH)–MYCN*–driven neuroblastoma mouse model presented by De Wyn *et al.* (*41*) and found that *MOXD1* expression steadily decreases with tumor progression (Fig. 3A). Mice without tumors (wild-type) maintained the *MOXD1* expression levels in the corresponding sympathetic ganglia above baseline over time (Fig. 3A).

To evaluate the sufficiency of loss of *MOXD1* to drive neuroblastomas, we knocked out *MOXD1* with CRISPR-Cas9 in the MES-dominant SH-EP cells that express high endogenous levels of *MOXD1* (Fig. 2E). We used the chorioallantoic membrane (CAM) assay. We implanted SH-EP *MOXD1* knockout (KO) and respective control cells at the bifurcation of an allantoid vein on the CAM in fertilized eggs in vivo at embryonic day 10 (E10; Fig. 3, B and C). We found that implantation of *MOXD1* knockout cells reduced the survival of embryos to 71% (*n* = 27 of 38) after 2 days (E12) of tumor growth and to as low as 5% (*n* = 2 of 38) after 4 days (E14; Fig. 3D) as compared to the lack of appreciable effect on survival of the host embryo until after 4 days (E14) and a decrease in viability to 29% (*n* = 8 of 28) with implantation of control cells (Fig. 3D). A substantially higher number of tumors were formed with *MOXD1* knockout cells (*n* = 20 of 27) at 2 days after implantation as compared to control cells (*n* = 10 of 28) (Fig. 3E). Knockout of *MOXD1* resulted in larger tumors (Fig. 3F). As visualized before tumor dissection, the *MOXD1* knockout cells were also more migratory (tumor and motile cells marked by dashed lines, Fig. 3G).

### Knockout of *MOXD1* increases tumor growth and penetrance in a neuroblastoma zebrafish model

**Fig. 4.**
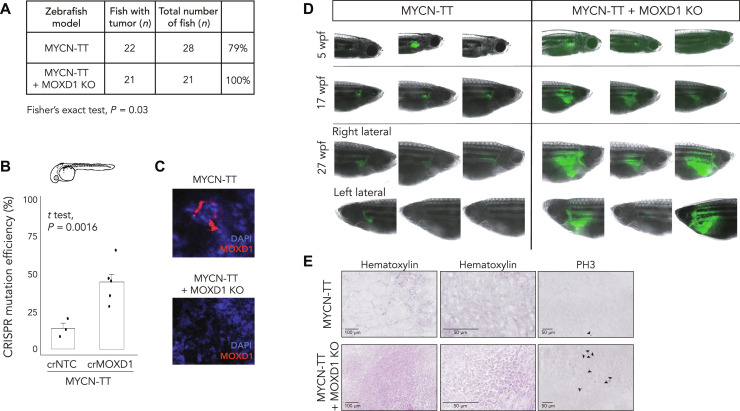
*MOXD1* knockout (KO) accelerates tumor penetrance in zebrafish. (**A**) Summary of MYCN-TT and MYCN-TT + MOXD1 KO zebrafish with tumors. *P* value by Fisher’s exact test as indicated. (**B**) CRISPR mutation efficiency in MYCN-TT + MOXD1 KO and MYCN-TT zebrafish as determined by a tumor sample analysis on Miseq and analyzed using CRISPResso2.0 software (http://crispresso2.pinellolab.org/submission). (**C**) Confirmation of CRISPR-Cas9–mediated knockout of MOXD1 at protein level in tumors dissected from MYCN-TT only and MYCN-TT + MOXD1 KO zebrafish. Staining of MOXD1 by immunofluorescence. DAPI was used to counterstain the nuclei. (**D**) epresentative images of zebrafish at 5, 17, and 27 wpf. At week 27, each fish was photographed from both sides (right and left lateral sides). Cancer cells were visualized by fluorescence. Note that the images are not from the corresponding fish at each time point, but randomly picked from the whole population. (**E**) Hematoxylin staining to visualize the tissue of MYCN-TT and MYCN-TT + MOXD1 KO zebrafish tumors as well as staining with phospho-Histone H3 (PH3) visualizing mitosis. Arrowheads denote mitotic cells.

We further investigated the impact of *MOXD1* on neuroblastoma tumorigenesis using the Tg(*dbh:MYCN*; *dbh:EGFP*) zebrafish model generated by Tao *et al.* (*42*), hereafter referred to as MYCN-TT (Fig. 4), which coexpresses enhanced green fluorescent protein (eGFP) and human MYCN under the control of the zebrafish *d*β*h* promoter, and found that there was a baseline tumor penetrance of 79% (Fig. 4A). We knocked out *MOXD1* with high CRISPR mutation efficiency (Fig. 4B) and ablated the MOXD1 protein levels, which we confirmed in tumors dissected at 27 weeks post-fertilization (wpf) (Fig. 4C). We found that *MOXD1* knockout during the embryonic stage increased the tumor penetrance to 100% (*n* = 21 of 21) (Fig. 4A). All MYCN-TT + MOXD1 KO fish developed tumors in the interrenal gland after 5 weeks. The analyses of the fish at 5, 17, and 27 wpf showed a remarkable difference in tumor size (visualized by fluorescence microscopy; Fig. 4D). Sectioning of the dissected tumors at 27 wpf revealed differences in tissue architecture, where the MYCN-TT–derived tumors comprised large amounts of connective tissue and contained smaller foci of cancer cells (Fig. 4E), while MYCN-TT + MOXD1 KO–derived tumors largely consisted of cancer cells (Fig. 4E). We also found that the proportion of mitotic cells [as measured by the phospho-Histone H3 (PH3) expression] was higher in *MOXD1* KO tumors (Fig. 4E).

### MOXD1 overexpression in ADRN-like cells prolongs survival and reduces tumor burden in in vivo mouse models

**Fig. 5.**
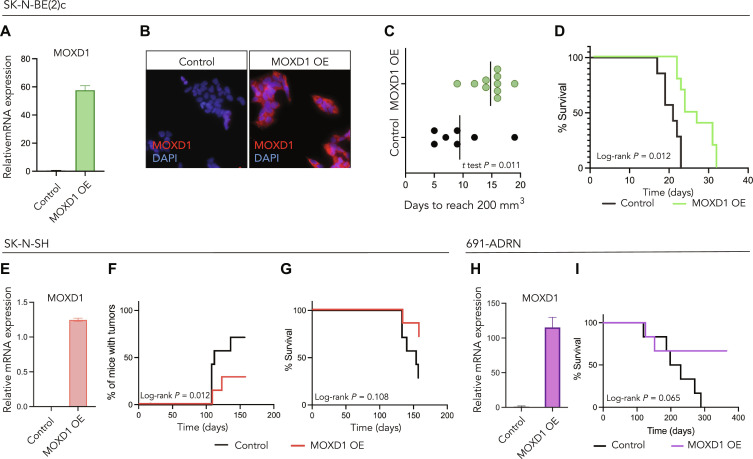
*MOXD1* overexpression in ADRN-like cells prolongs survival and reduces tumor burden in in vivo mouse models. (**A** and **B**) Confirmation of *MOXD1* overexpression by lentiviral transduction at the mRNA (A) and protein (B) level in neuroblastoma SK-N-BE(2)c cells by qPCR and immunofluorescence, respectively. DAPI was used to counterstain the nuclei. Error bars denote SD from *n* = 3 biologically independent repeats. (**C**) Time to tumor formation in mice subcutaneously injected with SK-N-BE(2)c cells transduced with an empty control vector or an MOXD1 overexpression (OE) vector determined by days to reach a volume of 200 mm^3^. The line indicates the mean and *P* value by Student’s *t* test as indicated (*n* = 10 in MOXD1 OE and *n* = 7 in CTRL). (**D**) Kaplan-Meier plot of the overall survival in mice of the indicated subgroup of SK-N-BE(2)c (*n* = 10 MOXD1 OE, *n* = 7 CTRL). *P* value by the log-rank (Mantel Cox) test as indicated. (**E**) Confirmation of *MOXD1* overexpression by lentiviral transduction at the mRNA level in neuroblastoma SK-N-SH cells by qPCR. Error bars denote SD from *n* = 3 biologically independent repeats. (**F**) Percentage of mice with tumors from the indicated subgroup of SK-N-SH cells (*n* = 7 per group). *P* value by log-rank (Mantel Cox) test. (**G**) Kaplan-Meier plot of overall survival in mice of the indicated subgroup of SK-N-SH (*n* = 7 per group). *P* value by the log-rank (Mantel Cox) test as indicated. (**H**) Confirmation of *MOXD1* overexpression at the mRNA level in neuroblastoma 691-ADRN cells by qPCR. Error bars denote SD from *n* = 3 biologically independent repeats. (**I**) Kaplan-Meier plot of survival in mice of the indicated subgroups of 691-ADRN cells (*n* = 6 per group). *P* value by the log-rank (Mantel Cox) test as indicated.

To evaluate the effect of *MOXD1* expression on tumorigenesis, we overexpressed *MOXD1* in three cell lines with an in vitro dominant ADRN phenotype [SK-N-BE(2)c, SK-N-SH, and 691-ADRN; Fig. 5], which, as we showed above, lack the expression of endogenous *MOXD1* (Fig. 2E) and verified this overexpression by quantitative polymerase chain reaction (qPCR) and immunofluorescence staining (Fig. 5, A, B, E, and H). We then injected SK-N-BE(2)c^CTRL^ and SK-N-BE(2)c^MOXD1-OE^ cells subcutaneously into the flank of nude mice and found that mice injected with *MOXD1*-overexpressing cells presented with delayed tumor formation with a mean time of 15 days [95% confidence interval (CI) 12.8 to 16.8] for tumors to reach a volume of 200 mm^3^ as compared to a mean time of 9 days (95% CI 4.9 to 14.0) for mice injected with empty vector control cells (Fig. 5C). The delay in tumor formation was reflected by prolonged survival (Fig. 5D). Further, we found that fewer mice injected with SK-N-SH^MOXD1-OE^ cells developed tumors (Fig. 5F) and that this group showed prolonged survival (Fig. 5G). Injection of 691-ADRN^MOXD1-OE^ cells, which carry overexpression of *MOXD1*, resulted in prolonged survival (Fig. 5I). Notably, all surviving mice in the 691-ADRN MOXD1 overexpressing (MOXD1 OE) group were tumor free at the experimental end point (365 days) as compared to the CTRL group in which all mice had tumors (Fig. 5I). Subsequently, we cultured SK-N-BE(2)c^CTRL^ and SK-N-BE(2)c^MOXD1-OE^ cells in a three-dimensional colony formation system and found no difference in the number of colonies formed between these cells (fig. S5, A and B); however, the cells overexpressing *MOXD1* formed smaller colonies (fig. S5C). No differences were observed in two-dimensional cell proliferation, Caspase-3 activity, neuronal differentiation, chemotherapy resistance, cell motility, or angiogenic capacity between the groups (fig. S5, D to M). Together, these results demonstrate that expression of *MOXD1* suppresses neuroblastoma tumorigenesis.

### * MOXD1* overexpression reveals enrichment in the pathways associated with tumor growth and embryonic development

**Fig. 6.**
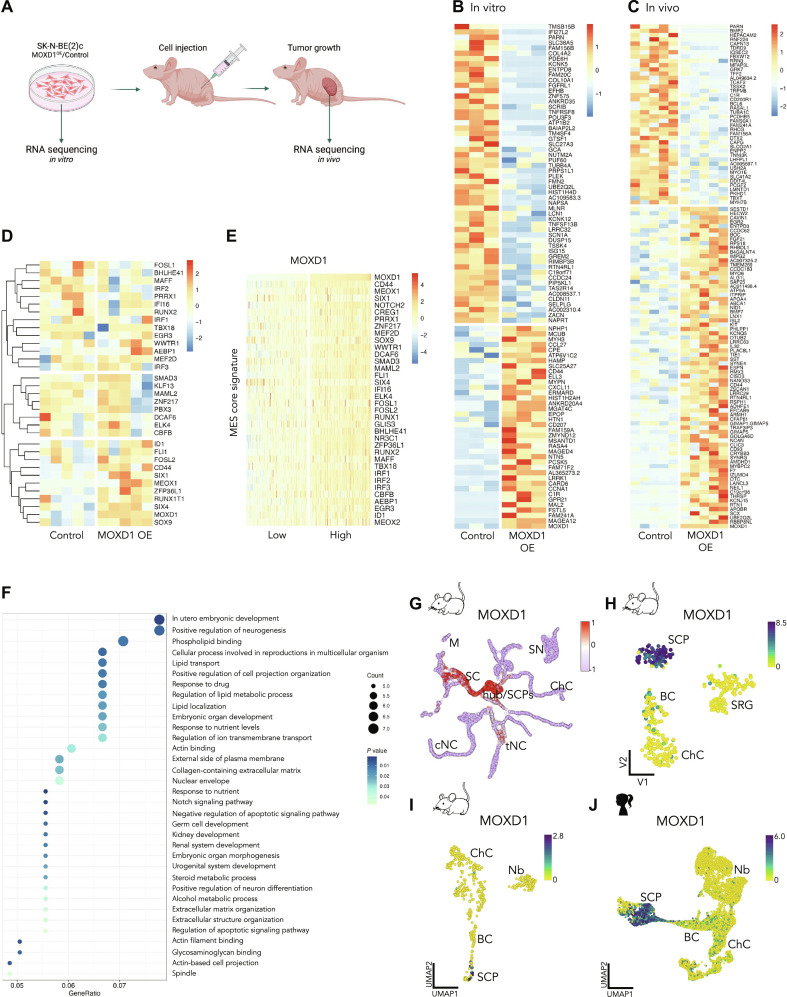
*MOXD1* enriches for pathways associated with embryonic development and its expression is restricted to SCPs. (**A**) Schematic illustration of in vitro grown SK-N-BE(2)c cells [MOXD1 overexpression (OE) or control] and in vivo tumors generated from the same in vitro grown SK-N-BE(2)c cells injected subcutaneously, collected for RNA-seq. Created with BioRender.com. (**B** and **C**) Heatmaps of the identified DEGs between wild-type (control) and MOXD1 OE SK-N-BE(2)c cells in vitro (B) and in vivo in mice (C). (**D**) Heatmap with unsupervised clustering analysis of MES core signature genes adapted from Thirant *et al.* (*16*) of the samples in (C). (**E**) Heatmap of MES core signature genes adapted from Thirant *et al.* (*16*) of the 25% of neuroblastomas with lowest *MOXD1* expression versus the 25% neuroblastomas with highest *MOXD1* expression in the SEQC cohort. (**F**) Gene ontology enrichment analysis of the DEGs in (C). (**G**) Mapping of *MOXD1* expression on single cells from mouse neural crest and Schwann cell linages from Kastriti *et al.* (*28*). (**H** and **J**) Visualization of *MOXD1* by cell-type clustering on a single-cell analysis of (H) mouse neural crest from Furlan *et al.* (*44*), (I) developing mouse adrenal medulla from Hanemaaijer *et al.* (*23*), and (J) human adrenal lineage from Jansky *et al.* (*24*). Color scale shows log_2_-transformed expression. BC, bridge cells; ChC, chromaffin cells; cNC, cranial neural crest; M, melanocytes; Nb, neuroblast; SC, Schwann cells; SCP, Schwann cell precursors; SN, sympathetic neurons; SRG, suprarenal sympathetic ganglion; tNC, trunk neural crest.

We performed RNA-seq on in vitro–cultured SK-N-BE(2)c^CTRL^ and SK-N-BE(2)c^MOXD1-OE^ cells and found 94 differentially expressed genes (DEGs) (Fig. 6, A and B). We then implanted the same in vitro grown cells into mice (tumor formation shown in Fig. 5C), resected the tumors at the experimental end point (volume >1800 mm^3^), and performed RNA-seq on these resected samples and retrieved a list of 117 DEGs (Fig. 6, A and C). We found that both datasets had enriched *MOXD1* expression in the samples from SK-N-BE(2)c^MOXD1-OE^ cells and their derived tumors, respectively, confirming the maintained overexpression during prolonged culture in vitro and growth in vivo (Fig. 6, B and C).

To build on the observed relationship between *MOXD1* expression and the MES phenotype (Fig. 2), we compared our in vivo RNA-seq data (Fig. 6C) to the MES core signature defined by Thirant *et al.* (*16*). We found three major clusters (Fig. 6D), where the cluster with enrichment in the MOXD1 OE samples contained genes such as *CD44*, *SOX9*, and *MEOX1*, in addition to *MOXD1*. However, a MOXD1-driven full switch to an MES phenotype seemed unlikely on the basis of the results that the two other clusters entailed great variation within the groups, with ambiguous expression pattern for other known MES genes such as *PRRX1* and *SMAD3*. To compare our in vivo MES signature with patient data, we separated the SEQC patient cohort into *MOXD1*^Low^ (first quartile) and *MOXD1*^High^ (fourth quartile) groups and observed that the MES core signature was enriched in *MOXD1*^High^ tumors (Fig. 6E).

Next, we performed a gene ontology analysis on the DEGs retrieved from the in vivo RNA-seq data (Fig. 6C), which revealed enrichment in genes connected to several processes involved in tumor growth, including the extracellular matrix and Notch signaling (Fig. 6F). Our analysis further highlighted genes involved in the regulation of ion transmembrane transport and intracellular localization (Fig. 6F), which is consistent with the previously suggested copper ion-binding function of *MOXD1* (*43*). At last, we observed an enrichment of genes involved in several processes connected to embryonic development (Fig. 6F).

### * MOXD1* is enriched in SCPs

Building on the enrichment of genes involved in embryonic development (Fig. 6, C and F) and the documented expression of *MOXD1* in trunk neural crest cells (*29*, *30*), we decided to analyze the expression pattern of *MOXD1* across various cell types during embryogenesis. Our analysis of publicly available scRNA-seq data from E9.5 to E12.5 mouse embryos previously presented by Kastriti *et al.* (*28*) showed an enrichment of *MOXD1* not only in trunk neural crest cells but also in Schwann cell precursors (SCPs; Fig. 6G). Further analyses of scRNA-seq data from precursor cells of the sympathoadrenal lineage during mouse development (*44*) and snRNA-seq data from mouse and human development (*23*, *24*) consistently validated that *MOXD1* expression was highly enriched in, and unique to, SCPs (Fig. 6, H to J). SCPs are multipotent nerve-associated neural crest–derived cells that can give rise to both immature sympathoblasts and chromaffin cells, cell types of which neuroblastomas are composed (*25*). The neuroblastoma MES gene signature has previously been shown to have pronounced overlap with the SCP cluster (*14*), further emphasizing the corresponding lineage-specific expression of *MOXD1* in both healthy and tumor development.

## DISCUSSION

Herein, we identified *MOXD1* as a de novo tumor-suppressor gene in neuroblastoma, effective by loss of the 6q23 locus surrounding this gene. We recapitulated these findings in multiple translational models, including mouse, chick, and zebrafish, and showed that this fundamental genetic regulatory step in neural crest cell fate and neuroblastoma cell phenotype is highly conserved along the vertebrate lineage.

We found that low expression level of *MOXD1* is an independent prognostic marker of poor outcome among patients with neuroblastoma of all stages and risk groups. In addition, individuals with loss of function of *MOXD1* had reduced survival rates compared to the rest of the cohort of neuroblastoma patients with high-risk disease, which already have a dismal prognosis. These results suggested a tumor-suppressing function of *MOXD1*. To investigate these clinical correlations, we introduced experimental knockout in vivo studies in chick embryos and zebrafish. Transplantation of neuroblastoma cells using the CAM assay allowed us to monitor tumor formation and tumor cell motility, while our zebrafish model, which is based on oncogene-induced neuroblastoma formation in the interrenal gland, allowed us to determine how additional knockout of *MOXD1* affected tumor penetrance, growth, and histology. We indeed found that *MOXD1* knockout increased tumor penetrance, tumor formation, and cell motility, leading to more aggressive disease. To further demonstrate the tumor-suppressing capacity of MOXD1, we set up the opposite experiment, i.e.*,* overexpressed the protein in neuroblastoma cells that completely lack endogenous expression. We transplanted these cells subcutaneously into mice to be able to monitor growth over time. The overexpression of *MOXD1* showed delay in tumor growth and increase in animal survival in three different neuroblastoma cell models. Our knockout and overexpression studies support that *MOXD1* acts as a tumor-suppressor gene in neuroblastoma.

Given our findings on MOXD1 in neuroblastoma, we evaluated expression of *MOXD1* in melanoma, which, like neuroblastoma, originates from trunk neural crest cells. We found that the *MOXD1* expression is higher in melanomas than in neuroblastomas and that the *MOXD1* levels are slightly higher in metastatic than in primary melanomas. Although neuroblastoma and melanoma are both trunk neural crest derived, they descend from cells that migrate along different pathways (ventral and dorsal, respectively) following delamination and EMT. A recent study on glioblastoma, a tumor form derived from neural crest derivatives other than the trunk, i.e.*,* with a separate origin from neuroblastoma, identified that high *MOXD1* expression is associated with a poor prognosis (*45*), opposite to our findings in neuroblastoma. To explore the prognostic role of *MOXD1* across cancer types of an origin separate from neural crest, we analyzed patient data from patients with breast and colon cancer. We observed that *MOXD1* does not discriminate between localized and aggressive diseases in any of these cancer types. Thus, we hypothesized that the role of *MOXD1* in cancer is tissue- and cell-specific, even depending on lineage commitment within the same embryonic progenitor cell population. This hypothesis was supported by our observation that *MOXD1* is expressed almost exclusively in SCPs, a subpopulation with a gene expression signature score correlating with a more favorable prognosis in neuroblastoma (*23*).

Recent studies have defined two distinct cellular phenotypes that foster neuroblastoma development and progression: immature MES and lineage-committed ADRN (*11*, *12*). These cell phenotypes are plastic and can convert into one another. The MES core gene signature as defined in Westerhout *et al.* (*14*), is highly enriched in SCPs. We found that *MOXD1* is expressed in MES- but not in ADRN-phenotypic cells, further supporting the hypothesis that *MOXD1* expression is lineage-restricted, specifically to SCPs during embryogenesis, and that the expression, or lack thereof, might affect the subtype and aggressiveness of the resulting tumor. Our RNA-seq data from both in vitro and in vivo grown cells identified the stem cell marker *CD44* as positively regulated by *MOXD1*. This cell surface protein has recently been identified as a strong marker for MES neuroblastoma cells (*16*). Expression of *CD44* was high in cells in which we overexpressed *MOXD1*, which is consistent with the previous finding that *CD44* is expressed in undifferentiated multipotent neural crest cells (*46*) and has been shown to be associated with favorable prognosis in neuroblastoma (*47*–*50*).

At this point, it is however unclear whether MOXD1 directs lineage commitment where loss of *MOXD1* skews cells toward a differentiated ADRN phenotype, or whether *MOXD1* is lost as a consequence of ADRN tipping. If loss of *MOXD1* is caused by altered lineage commitment, it will be of importance to understand how this is regulated at a mechanistic level, and the impact it potentially has on tumor initiation and progression. For example, identification of affected signal transduction, chromatin remodeling, or ion exchange, could open up new avenues for targeting neuroblastomas caused by loss of *MOXD1*.

Recent studies suggested that SCPs generate most chromaffin cells and a smaller portion of neuroblasts composing the adrenal medulla (*25*, *44*). However, a subsequent study debated whether these findings are true only in younger patients (younger than 18 months at diagnosis). Bedoya-Reina *et al.* (*17*) suggest that neuroblastoma in younger children with a better prognosis may arise from an SCP-derived subtype of neuroblastoma. This is consistent with our data showing expression of *MOXD1*, which we found to be associated with better prognosis in patients and increased survival in our translational models, is enriched in SCPs.

The sparse literature on MOXD1 functions suggests the involvement of copper binding (*43*). Disrupted copper homeostasis is known to be involved in the malignant progression of several tumor forms (*51*, *52*). Both increased and decreased levels of copper-related proteins are associated with cancer, and their use as predictive or prognostic biomarkers is promising (*53*). Our RNA-seq data indeed identified ion transport as one of the most affected processes connected to MOXD1, and, notably, we found that the copper ion–binding protein APOA4 is up-regulated with MOXD1 overexpression in tumor cells. We hypothesized that a putative MOXD1-APOA4 axis and thus functional and tightly regulated copper signaling is essential for embryonic neural crest homeostasis. Consistent with this theory, Poursani *et al.* (*54*) recently demonstrated a link between copper homeostasis and EMT in several cancer forms, including neuroblastoma (*54*). However, to fully unravel the mechanisms of MOXD1, additional studies with further functional and biological experiments are needed.

**Fig. 7.**
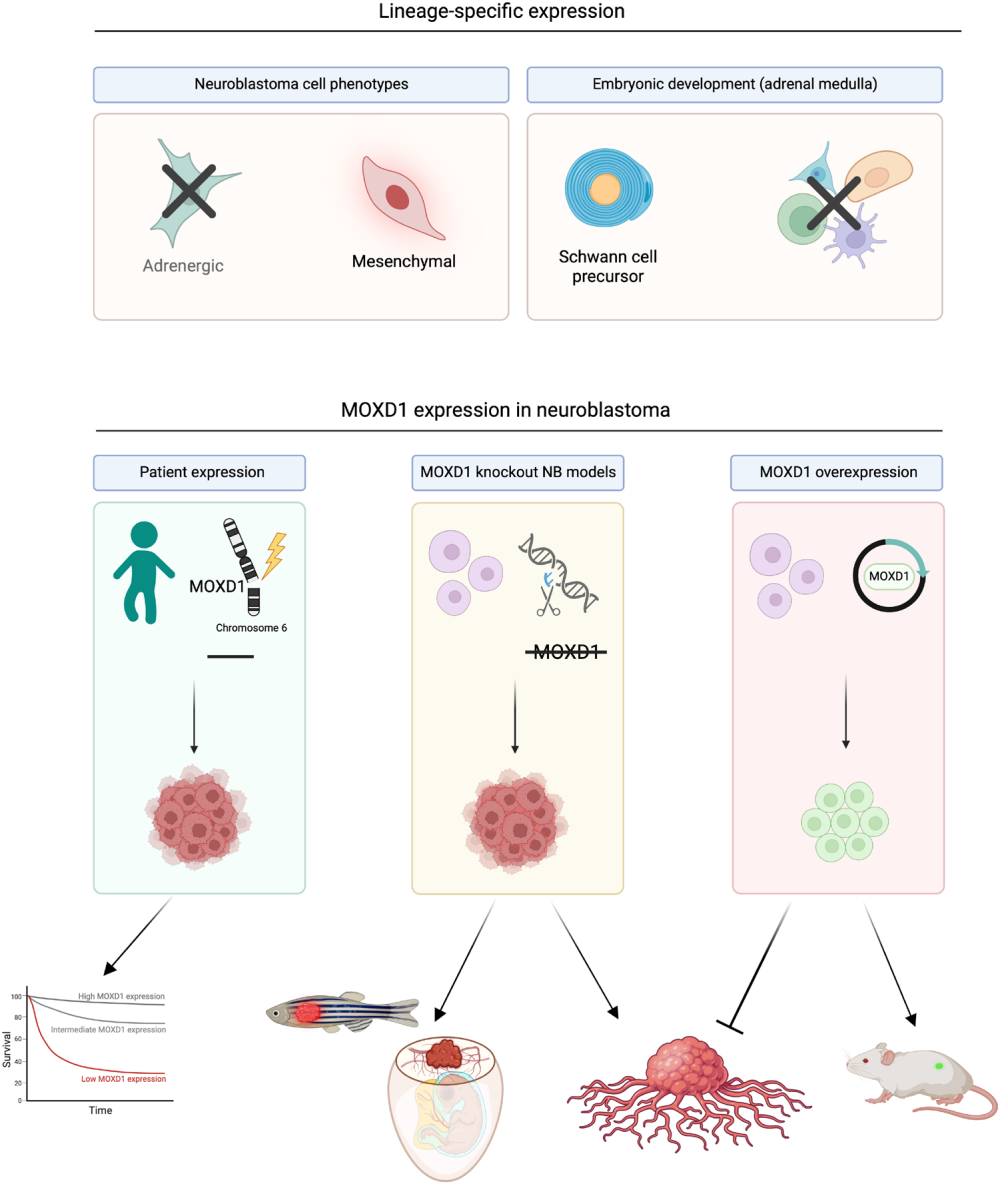
Graphical visualization of MOXD1 in healthy and tumor development. Created with BioRender.com.

In conclusion, we found that *MOXD1* is a tumor-suppressor gene in neuroblastoma. We further showed that MOXD1 discriminates between the ADRN and MES cell phenotypes, with cell-specific expression in the neural crest–like MES cells. In line with this, *MOXD1* expression was restricted to SCPs during normal development of human and mouse adrenal medulla, reflecting the overlap between normal SCPs and MES neuroblastoma cell phenotypes, which was previously reported (*14*). A graphical summary of our findings is presented in Fig. 7. Our results strengthen the hypothesis that neuroblastoma originates from stalled development during predefined time- and spatial-specific points. We depict a de novo tumor-suppressor gene and potentiates future identification of subtype-specific neuroblastomas. Our results suggest that such neuroblastoma subtypes might be derived from restricted cell lineages during healthy neural crest development, setting a framework for studies on subtype-specific treatment options.

## MATERIALS AND METHODS

### Ethics

According to Swedish regulation (Jordbruksverkets föreskrift L150, §5), experiments performed on chick embryos younger than E13 do not require ethical permit. All chick embryo and mouse procedures followed the guidelines set by the Malmö-Lund Ethics Committee for the use of laboratory animals and were conducted in accordance with the European Union directive on the subject of animal rights (ethical permit no. 18743/19). For the TMA, all patients, their relatives, or their legal guardians signed the appropriate written informed consent. The present study was approved by INCLIVA’s Clinical Research Ethics Committee (ref. 2017/198). All procedures involving zebrafish were approved by the local animal ethics committee (Ghent University hospital, Ghent, Belgium; ethical permit no. ECD 17/100) and performed according to local guidelines and policies in compliance with the national and European laws.

### Cell culture

The neuroblastoma cell lines SK-N-BE(2)c, SK-N-SH, SH-SY5Y, SH-EP, and IMR-32 were cultured in minimum essential medium (MEM) or RPMI 1640 (IMR-32), supplemented with 10% fetal bovine serum. The 691-ADRN cells were cultured in Dulbecco’s modified Eagle’s medium (DMEM)/F12 supplemented with fibroblast growth factor (40 ng/ml), epidermal growth factor (20 ng/ml), and 1 × B27 supplement. The MS1 mouse endothelial cells were cultured in DMEM supplemented with 10% fetal bovine serum. Penicillin (100 U) and streptomycin (10 μg/ml) were added to all cultures. Cells were kept in a humidified incubator at 37°C with 21% O_2_ and 5% CO_2_. SK-N-BE(2)c, SK-N-SH, SH-SY5Y, SH-EP, IMR-32, and MS1 were grown as a monolayer and dissociated using trypsin and accutase. In addition, 691-ADRN cells were grown in suspension and dissociated using trypsin. All cells were, at minimum, trimonthly screened for mycoplasma (Eurofins Genomics). The 691 isogenic pair (2018) and SK-N-BE(2)c, IMR-32, SH-SY5Y, and SH-EP cell lines (all 2022) were authenticated by STR profiling (Eurofins Genomics).

### Cloning

For CRISPR-Cas9 targeting, oligos designed to target *MOXD1* (MOXD1.4.gRNA Top oligo–5′ ggatgGCACCATGTGACAAAGgtg 3′, Bot oligo–5′ aaaccacCTTTGTCACATGGTGCc 3′) were annealed pairwise at a concentration of 100 μM per oligo using the T4 DNA ligase buffer by heating to 95°C for 5 min. The annealed oligo reactions were cooled to room temperature and diluted 1:1000. The U6.3 > gRNA.f + e (no. 99139, Addgene) vector was digested over night with BsaI-HF enzyme (New England Biolabs, no. R3733) and gel extracted. Guide RNAs (gRNAs) were cloned into the digested U6.3 > gRNA.f + e vector using T4 DNA ligase (New England Biolabs, no. M0202) at room temperature for 20 min. Successful inserts were identified by colony PCR using U6 sequencing primer and gRNA reverse oligo specific to *MOXD1* gRNA.

### Transfection of CRISPR-Cas9–knockout constructs

Transfection of SH-EP cells was performed using 5 μg of cloned MOXD1-targeting or nontargeting control gRNA constructs and Lipofectamine 3000 during an incubation time of 4 hours. Cells were either expanded in culture or seeded on coverslips for further immunofluorescence analysis. Knockout was verified by protein staining on a regular basis over the course of several weeks to ensure maintained absence of MOXD1.

### Lentiviral transduction

SK-N-BE(2)c, SK-N-SH, and ADRN-691 cells were transduced with a lentiviral vector encoding *MOXD1* gene or a control plasmid (OriGene, RC205862L2; PS100071). Lentiviruses were directly added to the cell culture media and incubated for 16 hours before media change.

### Soft agar colony formation

The SK-N-BE(2)c cells were plated at 2.5 × 10^3^ cells per well in six-well tissue culture plates with a mix of DMEM and 0.3% agar on top of a coating layer of 0.6% agar. The plates were incubated at 37°C in humidified atmosphere for 21 days. The colonies were stained with crystal violet (0.001%, Sigma-Aldrich, no. C0775). The colony number and size were automatically calculated with ImageJ. Four biologically independent replicates were used for quantification and statistical analysis.

### Cell viability and drug response

SK-N-BE(2)c cells were plated in technical triplicates in opaque 96-well plates with a total volume of 100 μl and thereafter incubated for 24 hours pretreatment to allow attachment to the plate. The cells were treated with the indicated logarithmic concentration ranges of cisplatin (Sigma-Aldrich, no. C2210000) and doxorubicin (Sigma-Aldrich, no. D1515) dissolved in sterile H_2_O. Cell viability was measured using CellTiter-Glo (Promega Corp., no. G7571). Three biologically independent replicates were used for quantification and statistical analysis.

### Cell proliferation and differentiation

SK-N-BE(2)c cells were seeded at 1× 10^5^ cells per well in a six-well plate, incubated for 5 days, and manually counted using a Bürker chamber. Four biologically independent replicates were used for quantification. For the differentiation assay, the cells were grown for 24 hours, and the number of neurites was manually counted by blinded quantification from representative images. The number of neurites were normalized to the total number of cells (i.e.*,* neurites per cell). Three biologically independent replicates were used for quantification.

### Active caspase-3 ELISA

To measure apoptosis, we used a human active caspase-3 ELISA kit (Invitrogen, no. KHO1091). Control and MOXD1 OE SK-N-BE(2)c cells were seeded and incubated for 24 hours to allow for attachment. The medium was then removed, and the cells were lysed with radioimmunoprecipitation assay buffer supplemented with complete protease inhibitor. Apoptotic activity was determined according to the manufacturer’s instructions. Activity measurements were performed in triplicates.

### Wound healing

Cells were seeded at 3 × 10^5^ cells per well in a six-well plate and incubated at 37°C for 24 hours (for the cells to reach 80% confluency) before a scratch or “wound” was made in the middle of the culture with a p1250 pipette tip. The images were captured at 0, 24, 48, and 72 hours after the scratch. Four biologically independent replicates were used for quantification. Open areas were manually marked in ImageJ and then automatically measured.

### Coculture angiogenesis

MS1 (pancreatic islet mouse endothelial) cells (10^4^) were mixed with SK-N-BE(2)c cells (1,1 ratio) and seeded in a 96-well plate on top of a layer of growth factor–reduced Matrigel (BD Biosciences, no. 354230) in 1:2 MEM and DMEM (1,1). Matrigel was thawed at 4°C overnight, added to 96-well plates, and transferred to 37°C for polymerization before seeding. Cells were incubated for 8 hours before four images of each group were captured. The images were analyzed in ImageJ using the Angiogenesis Analyzer plug-in.

### Immunofluorescence

Transfected SH-EP for knockout, and transduced SK-N-BE(2)c for overexpression, cells were seeded on coverslips and allowed to attach overnight. Cells were fixed in 4% paraformaldehyde for 15 min. Coverslips were washed in phosphate-buffered saline (PBS) before blocking in 3% bovine serum albumin and 0.3% Triton-X in PBS for 1 hour. Slides were then incubated with primary antibody (MOXD1, Thermo Fisher Scientific, see details in table S1) for 1 hour at room temperature. Coverslips were washed, and secondary antibody mixed with 4′,6-diamidino-2-phenylindole (DAPI) was added for 1 hour at room temperature. Coverslips were finally mounted on slides and imaged.

Zebrafish tumor sections were blocked in 5% goat serum and 0.3% Triton-X for 1 hour at room temperature before incubation with primary antibody (MOXD1, Aviva; see details in table S1) at +4°C overnight. Slides were washed in PBS before incubation with a mix of secondary antibody and DAPI for 1 hour at room temperature. Coverslips were mounted on slides and imaged.

### Immunohistochemistry

Slides were immersed in hematoxylin for 5 min, dehydrated in incremental concentrations of ethanol (50, 70, 80, 100, and 100%) for 3 min each, immersed in xylene twice for 5 min each, and finally mounted with Eukitt mounting media.

To stain for mitotic cells, slides were washed in PBS and then treated with 3% hydrogen peroxide solution for 45 min. After rinse and wash in PBS-Tween (PBS-T), slides were incubated with blocking solution for 1 hour at room temperature. Slides were incubated with primary antibody (PH3; see details in table S1) at +4°C overnight, washed in PBS-T, and incubated with secondary antibody for 1 hour at room temperature. Slides were again washed and then incubated with streptavidin peroxidase for 2 hours. After rinse in PBS, the peroxidase reaction product was visualized with 0.05% 3,3′diaminobenzidine tetrahydrochloride (DAB) and 0.025% hydrogen peroxide in PBS for 10 min at room temperature. The reaction was stopped using PBS, and slides were mounted before imaging.

### RNA extraction and quantitative real-time PCR

Total RNA from the cells was extracted using RNAqueous Micro Kit (Ambion RNAqueous-Micro Kit, Thermo Fisher Scientific, no. AM1931) and eluted in 20-μl elution solution. Total RNA from the tumors was extracted using QIAshredder (Qiagen, catalog no. 79656) and RNeasy Midi Kit (Qiagen, catalog no. 75142) and eluted in 30-μl ribonuclease–free water. cDNA synthesis was performed using random primers (Applied Biosystems Reverse Transcriptase Kit, Thermo Fisher Scientific, no. 4368814). Quantitative reverse transcription PCR was performed using the SYBR Green PCR Master Mix (Thermo Fisher Scientific, no. 4364346). The relative mRNA expression was normalized to the expression of three reference genes (*UBC*, *YWHAZ*, and *SDHA*) using the comparative C_t_ method (*55*). The primers are listed in table S2.

### Tissue microarray

Together, 50 high-risk neuroblastoma samples with *MYCN* amplification were analyzed in a TMA, consisting of one 1-mm cylinder from each of the 50 high-risk neuroblastomas with *MYCN* amplification. The patient samples included in the TMA were referred to the Spanish Reference Centre for Neuroblastoma Biological and Pathological studies (Department of Pathology, University of Valencia-INCLIVA) between 1999 and 2017. Patient data were collected by the pediatric oncologists in charge at the hospital of origin and by the clinicians of the Reference Centre for neuroblastoma clinical studies and were classified according to the INRG pretreatment stratification criteria.

Paraffin-embedded TMAs were sectioned (3 μm) and stained for MOXD1 using a DAKO Cytomation Autostainer Plus with the EnVision FLEX high pH kit (Agilent, code K8010). The slides were dried at 60°C for 1 hour, paraffin was removed, and the slides were placed in a pressure cooker for 20 min at 97°C in the EnVision FLEX target retrieval solution low pH (pH 6; Agilent, code K8005). Then, the slides were washed in wash buffer (Agilent, code K8007); then, the EnVision FLEX peroxidase-blocking reagent (Agilent, code DM821) was added. The slides were incubated for 5 min and then washed as described above. The primary antibody was diluted in EnVision FLEX antibody diluent (Agilent, code K8006), and the slides were incubated for 30 min after washing as described above. The slides were then incubated in EnVision FLEX/HRP (Agilent, code DM822), washed as described above, and further incubated in EnVision FLEX DAB + chromogen (Agilent, code DM827) diluted in EnVision FLEX substrate buffer (Agilent, code DM823). After the last washing step, the slides were incubated with HTX for 3 min, dehydrated, and mounted.

The sections were digitalized with the PannoramicMIDI 3DHistech scanner at 20× magnification. Cytoplasmic brown staining was considered to indicate MOXD1 positivity. The number of MOXD1-positive cells and staining intensity was evaluated with the NuclearQuant module of Pannoramic Viewer software (3DHistech). This module could be applied to nonnuclear stained compartments by adjusting the nucleus size settings to detect the entire cells when required (nuclear radius 3 to 15 μm) and adjusting the color deconvolution settings. The detected artifacts and folded and/or broken regions were considered uninformative and were excluded from the image analysis. The automatically obtained results were validated by optical microscopy by a pathologist and two expert researchers in morphology. The percentage of positive cells in each sample for every MOXD1 intensity expression (low, medium, and high) was calculated. For statistical purposes, we defined samples as low (≤25th percentile) or high (>25th percentile) MOXD1 expressions, according to the percentage of positive cells digitally detected as presenting high-intensity MOXD1 expression. In addition, the homogeneous and heterogeneous MOXD1 samples were differentiated, and we considered a sample heterogeneous when its total MOXD1-positive cell population was characterized by cells with low-, medium-, and high-intensity MOXD1 expressions, but none of the positive cell subpopulations accounted for >66% of the total MOXD1-positive cell population. SPSS version 26 was used to perform the statistical analysis, setting the significance level at 95%. Using the chi-square test, we evaluated the statistical correlation between the MOXD1 expression patterns and patients’ age (<18 versus ≥18 months). The other INRG prognostic variables could not be tested because of the homogeneous characteristics of the studied cohort.

### In vivo tumorigenicity

Female athymic mice (NMRI-Nu/Nu strain; Taconic) were housed in a controlled environment. Together, 0.8 × 10^6^ SK-N-BE(2)c, 1 × 10^6^ SK-N-SH, or 0.7 × 10^6^ 691-ADRN cells were subcutaneously injected with a 100-μl 2:1 mixture of cell culture media and growth factor–reduced Matrigel (BD, 354230) to the right flank of the mice. Mice were euthanized when the tumors reached a diameter of >1800 mm^3^ or 12 months after the start of the experiment (end point, 365 days). Tumor pieces were snap-frozen and further processed for RNA-seq.

### CAM assay

Fertilized eggs were stored at 37.5°C in humidified incubator. The eggs were windowed at E7, cells implanted at E10, and tumors harvested at E14. One million SH-EP cells (*MOXD1* knockout or CTRL) were suspended in a mix of Matrigel (Corning, no. 354234) and media (1:3) and grafted close to the bifurcation of an allantoid vein. There was no interference with the embryo. The eggs were sealed with an opaque tape and returned to the incubator. Ringer’s solution was added at E12 to keep the embryos moist. At harvest, tumors were photographed, the viability of embryos was assessed, and the tumors were analyzed by size and weight.

### Zebrafish models

Zebrafish were maintained in a Zebtec semi-closed recirculation housing system (Techniplast, Italy). The water had a constant temperature (27° to 28°C), pH (~7.5), conductivity (~500 μS), and light/dark cycle (14/10 hours). The fish were fed twice a day with dry food (Gemma Micro, UK) and once with Artemia (Ocean Nutrition, Belgium).

#### Determining the best crRNA for MOXD1-KO

CRISPR RNAs (crRNAs) targeting *MOXD1* were designed and injected according to a previously published workflow (*56*). First, the online Benchling tool (https://benchling.com/crispr) was used to select five MOXD1 crRNAs (CATGTTGGGATGTCAATAGG, CCAGGCATGACGGATTACAT, GATGCTGGAGTCATCGAGAC, GAGGCGCTACGATGCTGGAG, and CGCCAAGTGCGAGAGTTTAC) with the highest on-target score (efficiency score) and lowest off-target (specificity score) scores. These crRNAs (200 μM) were mixed together with a general tracrRNA (200 μM) (IDT, no. 1072533) to generate five different gRNA duplexes. Subsequently, 900-pg gRNA duplex was combined with 900-pg Cas9 enzyme (IDT, no. 1081061) before injection (1.4 nl). A nontargeting crRNA (GCAGGCAAAGAATCCCTGCC) was used as a control. The injections of all five crRNA:tracrRNA-Cas9 mixtures were performed in the yolk of wild-type zebrafish embryos during the one-cell stage. For each crRNA injection mix, DNA was isolated from a pool of 24 to 48 hours post-fertilization (hpf) embryos and amplified using specific primers surrounding the targeted region. The amplified pooled DNA sample was run on a Miseq and analysed using CRISPResso2.0 to determine the mutation efficiency. crMOXD1_3 (GATGCTGGAGTCATCGAGAC) resulted in the highest efficiency score and was used in subsequent experiments.

#### Injection of crMOXD1 in MYCN-TT [Tg(dβh:eGFP;dβh:MYCN)] zebrafish line

The CRISPR-Cas9 injection mixes of crRNA_MOXD1_3 and negative control were injected in MYCN-TT one-cell stage zygotes. This transgenic zebrafish line was previously established by coinjection of eGFP and human MYCN, both under the control of the zebrafish DBH promotor, which allowed the selection of MYCN-TT–positive larvae at 5 days after fertilization and the detection of neuroblastoma formation by eGFP expression in the interrenal gland of the zebrafish. From 5 wpf, the zebrafish were screened for tumor formation biweekly by fluorescence microscopy (Nikon, SMZ18).

The mutation efficiency of the injections was checked on Miseq in a separate embryo pool (24 to 48 hpf), and the experiments were only continued when the mutation efficiencies were higher than 60%. At 9 wpf, genotyping was performed by fin-clipping to confirm the MYCN-TT–positive genotype. Only the MYCN-TT–positive zebrafish were included in the study.

Tumors were harvested at 27 wpf (*n* = 6 crMOXD1, *n* = 3 crNTC), fixed in modified Davidson’s fixative [22% formaldehyde (37%), 12% glacial acetic acid, 33% ethanol (95%), and 34% distilled water] over night on shaker at room temperature and stored at −80°C until embedding. Tumor pieces were then directly embedded in gelatin, snap-frozen, and sectioned at 8 μm before degelatinized and processed for immunofluorescence and immunohistochemistry, as described above.

### RNA sequencing

RNA was extracted from the harvested cells in the culture (*n* = 3 biologically independent samples per group) or pieces from the in vivo grown tumors (*n* = 5 biologically independent samples per group). Sequencing was performed using NovaSeq 6000 (Illumina). The alignment of reads was performed using the HISAT2 software, and the reference genome was from the Ensemble database [Human GRCh38, GTF 94 (in vitro) or 99 (in vivo)]. The expression counts were performed using the StringTie software, and DEG analysis was performed using DESeq2.

The DEGs between the MOXD1 OE SK-N-BE(2)c cells and CTRL group were identified by a log_2_foldchange >1.5 and a higher expression in all samples in one group as compared to all the samples in the other group (in vitro) and log_2_foldchange >1.5 and a higher expression in >3 samples in one group as compared to all the samples in the other group (in vivo). Gene ontology enrichment analysis of the DEGs in vivo was performed with the R package clusterProfiler (*57*).

### Acquisition and analysis of datasets

The neuroblastoma patient datasets SEQC (GEO:GSE62564), Kocak (GEO:GSE45547), NRC (GEO:GSE85047), Depuydt (GEO:GSE103123), and melanoma (GEO:GSE65904), and the cell line datasets from van Groningen *et al.* (*12*) (R2 ID: ps_avgpres_gsenatgen2017geo52_u133p2), Boeva *et al.* (*11*) (GEO:GSE90683), Harenza *et al.* (*40*) (GEO:GSE89413), and the Cancer Cell Line Encyclopedia (GEO:GSE36133) were downloaded from the R2: genomics analysis and visualization platform (http://r2.amc.nl). The NB/GN (GEO:GSE7529), breast cancer (GEO:GSE102484), and colorectal cancer (GEO:GSE39582) datasets were downloaded from GEO. The data from Gartlgruber *et al.* (*15*) (GEO:GSE136209) were assessed from https://nbseb087.dkfz.de/project_NB_SE_viz/.

The RNA from the isogenic-pair dataset was provided by J. Koster, where RNA was extracted using a ribodepletion kit. FASTQ files were mapped to HG19 using HISAT2 and subsequently converted into gene expression values using cufflinks (genes mode) on UCSC genes. Last, the data were normalized by TPM.

 Survival analysis was performed by log-rank tests with the Kaplan-Meier method and multivariate Cox regressions using the R packages Survival and Survminer (Fig. 1, A and F, and figs. S1, A to G and S2C). Figure S2 (D to F) was generated using https://padpuydt.shinyapps.io/check_cn_in_hr_nb/, and the maximum size of aberration was set to 180 Mb. Further analysis of MOXD1 expression in the datasets was conducted in RStudio. The snRNA-seq data by Bedoya-Reina *et al.* (*17*) and the scRNA-seq data from Kastriti *et al.* (*28*) were analyzed using Pagoda at https://www.synapse.org/Synapse:syn22302605/wiki/604951 and https://adameykolab.srv.meduniwien.ac.at/glia_gene_umap/, whereas the scRNA-seq data from Furlan *et al.* (*44*), Gartlgruber *et al.* (*15*), Hanemaaijer *et al.* (*23*), and Jansky *et al.* (*24*) were analyzed using the R2 platform.

## Supplementary Material

20240621-1

## Notes

**Correction (9 July 2024):** Due to a production error, the labels for the subimages in Fig. 3G were blank. The figure has been updated in the online and PDF versions of the article to include the correct labels for each subimage.

## References

[1] K. K. Matthay, J. M. Maris, G. Schleiermacher, A. Nakagawara, C. L. Mackall, L. Diller, W. A. Weiss, Neuroblastoma. Nat. Rev. Dis. Primer 2, 16078 (2016). 10.1038/nrdp.2016.78 27830764

[2] J. M. Maris, Recent advances in neuroblastoma. N. Engl. J. Med. 362, 2202–2211 (2010). 20558371 10.1056/NEJMra0804577 PMC3306838

[3] G. M. Brodeur, Neuroblastoma: Biological insights into a clinical enigma. Nat. Rev. Cancer 3, 203–216 (2003). 12612655 10.1038/nrc1014

[4] N.-K. V. Cheung, M. A. Dyer, Neuroblastoma: Developmental biology, cancer genomics and immunotherapy. Nat. Rev. Cancer 13, 397–411 (2013). 23702928 10.1038/nrc3526 PMC4386662

[5] J. R. Park, S. G. Kreissman, W. B. London, A. Naranjo, S. L. Cohn, M. D. Hogarty, S. C. Tenney, D. Haas-Kogan, P. J. Shaw, J. M. Kraveka, S. S. Roberts, J. D. Geiger, J. J. Doski, S. D. Voss, J. M. Maris, S. A. Grupp, L. Diller, Effect of tandem autologous stem cell transplant vs single transplant on event-free survival in patients with high-risk neuroblastoma: A randomized clinical trial. JAMA 322, 746–755 (2019). 31454045 10.1001/jama.2019.11642 PMC6714031

[6] R. Ladenstein, U. Pötschger, A. D. J. Pearson, P. Brock, R. Luksch, V. Castel, I. Yaniv, V. Papadakis, G. Laureys, J. Malis, W. Balwierz, E. Ruud, P. Kogner, H. Schroeder, A. F. de Lacerda, M. Beck-Popovic, P. Bician, M. Garami, T. Trahair, A. Canete, P. F. Ambros, K. Holmes, M. Gaze, G. Schreier, A. Garaventa, G. Vassal, J. Michon, D. Valteau-Couanet, SIOP Europe Neuroblastoma Group (SIOPEN), Busulfan and melphalan versus carboplatin, etoposide, and melphalan as high-dose chemotherapy for high-risk neuroblastoma (HR-NBL1/SIOPEN): An international, randomised, multi-arm, open-label, phase 3 trial. Lancet Oncol. 18, 500–514 (2017). 28259608 10.1016/S1470-2045(17)30070-0

[7] M. S. Irwin, A. Naranjo, F. F. Zhang, S. L. Cohn, W. B. London, J. M. Gastier-Foster, N. C. Ramirez, R. Pfau, S. Reshmi, E. Wagner, J. Nuchtern, S. Asgharzadeh, H. Shimada, J. M. Maris, R. Bagatell, J. R. Park, M. D. Hogarty, Revised neuroblastoma risk classification system: A report from the children’s oncology group. J. Clin. Oncol. Off. J. Am. Soc. Clin. Oncol. 39, 3229–3241 (2021). 10.1200/JCO.21.00278 PMC8500606 34319759

[8] P. Depuydt, V. Boeva, T. D. Hocking, R. Cannoodt, I. M. Ambros, P. F. Ambros, S. Asgharzadeh, E. F. Attiyeh, V. Combaret, R. Defferrari, M. Fischer, B. Hero, M. D. Hogarty, M. S. Irwin, J. Koster, S. Kreissman, R. Ladenstein, E. Lapouble, G. Laureys, W. B. London, K. Mazzocco, A. Nakagawara, R. Noguera, M. Ohira, J. R. Park, U. Pötschger, J. Theissen, G. P. Tonini, D. Valteau-Couanet, L. Varesio, R. Versteeg, F. Speleman, J. M. Maris, G. Schleiermacher, K. De Preter, Genomic amplifications and distal 6q loss: Novel markers for poor survival in high-risk neuroblastoma patients. JNCI J. Natl. Cancer Inst. 110, 1084–1093 (2018). 29514301 10.1093/jnci/djy022 PMC6186524

[9] M. Rothstein, D. Bhattacharya, M. Simoes-Costa, The molecular basis of neural crest axial identity. Dev. Biol. 444, S170–S180 (2018). 30071217 10.1016/j.ydbio.2018.07.026 PMC6355384

[10] R. Soldatov, M. Kaucka, M. E. Kastriti, J. Petersen, T. Chontorotzea, L. Englmaier, N. Akkuratova, Y. Yang, M. Häring, V. Dyachuk, C. Bock, M. Farlik, M. L. Piacentino, F. Boismoreau, M. M. Hilscher, C. Yokota, X. Qian, M. Nilsson, M. E. Bronner, L. Croci, W.-Y. Hsiao, D. A. Guertin, J.-F. Brunet, G. G. Consalez, P. Ernfors, K. Fried, P. V. Kharchenko, I. Adameyko, Spatiotemporal structure of cell fate decisions in murine neural crest. Science 364, eaas9536 (2019). 31171666 10.1126/science.aas9536

[11] V. Boeva, C. Louis-Brennetot, A. Peltier, S. Durand, C. Pierre-Eugène, V. Raynal, H. C. Etchevers, S. Thomas, A. Lermine, E. Daudigeos-Dubus, B. Geoerger, M. F. Orth, T. G. P. Grünewald, E. Diaz, B. Ducos, D. Surdez, A. M. Carcaboso, I. Medvedeva, T. Deller, V. Combaret, E. Lapouble, G. Pierron, S. Grossetête-Lalami, S. Baulande, G. Schleiermacher, E. Barillot, H. Rohrer, O. Delattre, I. Janoueix-Lerosey, Heterogeneity of neuroblastoma cell identity defined by transcriptional circuitries. Nat. Genet. 49, 1408–1413 (2017). 28740262 10.1038/ng.3921

[12] T. van Groningen, J. Koster, L. J. Valentijn, D. A. Zwijnenburg, N. Akogul, N. E. Hasselt, M. Broekmans, F. Haneveld, N. E. Nowakowska, J. Bras, C. J. M. van Noesel, A. Jongejan, A. H. van Kampen, L. Koster, F. Baas, L. van Dijk-Kerkhoven, M. Huizer-Smit, M. C. Lecca, A. Chan, A. Lakeman, P. Molenaar, R. Volckmann, E. M. Westerhout, M. Hamdi, P. G. van Sluis, M. E. Ebus, J. J. Molenaar, G. A. Tytgat, B. A. Westerman, J. van Nes, R. Versteeg, Neuroblastoma is composed of two super-enhancer-associated differentiation states. Nat. Genet. 49, 1261–1266 (2017). 28650485 10.1038/ng.3899

[13] T. van Groningen, N. Akogul, E. M. Westerhout, A. Chan, N. E. Hasselt, D. A. Zwijnenburg, M. Broekmans, P. Stroeken, F. Haneveld, G. K. J. Hooijer, C. D. Savci-Heijink, A. Lakeman, R. Volckmann, P. van Sluis, L. J. Valentijn, J. Koster, R. Versteeg, J. van Nes, A NOTCH feed-forward loop drives reprogramming from adrenergic to mesenchymal state in neuroblastoma. Nat. Commun. 10, 1530 (2019). 30948783 10.1038/s41467-019-09470-w PMC6449373

[14] E. M. Westerhout, M. Hamdi, P. Stroeken, N. E. Nowakowska, A. Lakeman, J. van Arkel, N. E. Hasselt, B. Bleijlevens, N. Akogul, F. Haneveld, A. Chan, P. van Sluis, D. Zwijnenburg, R. Volckmann, C. J. M. van Noesel, I. Adameyko, T. van Groningen, J. Koster, L. J. Valentijn, J. van Nes, R. Versteeg, Mesenchymal-type neuroblastoma cells escape ALK inhibitors. Cancer Res. 82, 484–496 (2022). 34853072 10.1158/0008-5472.CAN-21-1621

[15] M. Gartlgruber, A. K. Sharma, A. Quintero, D. Dreidax, S. Jansky, Y.-G. Park, S. Kreth, J. Meder, D. Doncevic, P. Saary, U. H. Toprak, N. Ishaque, E. Afanasyeva, E. Wecht, J. Koster, R. Versteeg, T. G. P. Grünewald, D. T. W. Jones, S. M. Pfister, K.-O. Henrich, J. van Nes, C. Herrmann, F. Westermann, Super enhancers define regulatory subtypes and cell identity in neuroblastoma. Nat. Cancer 2, 114–128 (2021). 35121888 10.1038/s43018-020-00145-w

[16] C. Thirant, A. Peltier, S. Durand, A. Kramdi, C. Louis-Brennetot, C. Pierre-Eugène, M. Gautier, A. Costa, A. Grelier, S. Zaïdi, N. Gruel, I. Jimenez, E. Lapouble, G. Pierron, D. Sitbon, H. J. Brisse, A. Gauthier, P. Fréneaux, S. Grossetête, L. G. Baudrin, V. Raynal, S. Baulande, A. Bellini, J. Bhalshankar, A. M. Carcaboso, B. Geoerger, H. Rohrer, D. Surdez, V. Boeva, G. Schleiermacher, O. Delattre, I. Janoueix-Lerosey, Reversible transitions between noradrenergic and mesenchymal tumor identities define cell plasticity in neuroblastoma. Nat. Commun. 14, 2575 (2023). 37142597 10.1038/s41467-023-38239-5 PMC10160107

[17] O. C. Bedoya-Reina, W. Li, M. Arceo, M. Plescher, P. Bullova, H. Pui, M. Kaucka, P. Kharchenko, T. Martinsson, J. Holmberg, I. Adameyko, Q. Deng, C. Larsson, C. C. Juhlin, P. Kogner, S. Schlisio, Single-nuclei transcriptomes from human adrenal gland reveal distinct cellular identities of low and high-risk neuroblastoma tumors. Nat. Commun. 12, 5309 (2021). 34493726 10.1038/s41467-021-24870-7 PMC8423786

[18] O. C. Bedoya-Reina, S. Schlisio, Chromaffin cells with sympathoblast signature: Too similar to keep apart? Cancer Cell 39, 134–135 (2021). 33385330 10.1016/j.ccell.2020.12.009

[19] R. Dong, R. Yang, Y. Zhan, H.-D. Lai, C.-J. Ye, X.-Y. Yao, W.-Q. Luo, X.-M. Cheng, J.-J. Miao, J.-F. Wang, B.-H. Liu, X.-Q. Liu, L.-L. Xie, Y. Li, M. Zhang, L. Chen, W.-C. Song, W. Qian, W.-Q. Gao, Y.-H. Tang, C.-Y. Shen, W. Jiang, G. Chen, W. Yao, K.-R. Dong, X.-M. Xiao, S. Zheng, K. Li, J. Wang, Single-cell characterization of malignant phenotypes and developmental trajectories of adrenal neuroblastoma. Cancer Cell 38, 716–733.e6 (2020). 32946775 10.1016/j.ccell.2020.08.014

[20] Z. Liu, C. J. Thiele, Unraveling the enigmatic origin of neuroblastoma. Cancer Cell 38, 618–620 (2020). 33171126 10.1016/j.ccell.2020.10.016

[21] R. Yang, W. Luo, Y. Zhan, K. Li, J. Wang, R. Dong, Response to Kildsiute et al. and Bedoya-Reina and Schlisio. Cancer Cell 39, 136–137 (2021). 33385329 10.1016/j.ccell.2020.12.015

[22] H. Rohrer, Linking human sympathoadrenal development and neuroblastoma. Nat. Genet. 53, 593–594 (2021). 33833455 10.1038/s41588-021-00845-8

[23] E. S. Hanemaaijer, T. Margaritis, K. Sanders, F. L. Bos, T. Candelli, H. Al-Saati, M. M. van Noesel, F. A. G. Meyer-Wentrup, M. van de Wetering, F. C. P. Holstege, H. Clevers, Single-cell atlas of developing murine adrenal gland reveals relation of Schwann cell precursor signature to neuroblastoma phenotype. Proc. Natl. Acad. Sci. U.S.A. 118, e2022350118 (2021). 33500353 10.1073/pnas.2022350118 PMC7865168

[24] S. Jansky, A. K. Sharma, V. Körber, A. Quintero, U. H. Toprak, E. M. Wecht, M. Gartlgruber, A. Greco, E. Chomsky, T. G. P. Grünewald, K.-O. Henrich, A. Tanay, C. Herrmann, T. Höfer, F. Westermann, Single-cell transcriptomic analyses provide insights into the developmental origins of neuroblastoma. Nat. Genet. 53, 683–693 (2021). 33767450 10.1038/s41588-021-00806-1

[25] P. Kameneva, A. V. Artemov, M. E. Kastriti, L. Faure, T. K. Olsen, J. Otte, A. Erickson, B. Semsch, E. R. Andersson, M. Ratz, J. Frisén, A. S. Tischler, R. R. de Krijger, T. Bouderlique, N. Akkuratova, M. Vorontsova, O. Gusev, K. Fried, E. Sundström, S. Mei, P. Kogner, N. Baryawno, P. V. Kharchenko, I. Adameyko, Single-cell transcriptomics of human embryos identifies multiple sympathoblast lineages with potential implications for neuroblastoma origin. Nat. Genet. 53, 694–706 (2021). 33833454 10.1038/s41588-021-00818-x PMC7610777

[26] P. Kameneva, V. I. Melnikova, M. E. Kastriti, A. Kurtova, E. Kryukov, A. Murtazina, L. Faure, I. Poverennaya, A. V. Artemov, T. S. Kalinina, N. V. Kudryashov, M. Bader, J. Skoda, P. Chlapek, L. Curylova, L. Sourada, J. Neradil, M. Tesarova, M. Pasqualetti, P. Gaspar, V. D. Yakushov, B. I. Sheftel, T. Zikmund, J. Kaiser, K. Fried, N. Alenina, E. E. Voronezhskaya, I. Adameyko, Serotonin limits generation of chromaffin cells during adrenal organ development. Nat. Commun. 13, 2901 (2022). 35614045 10.1038/s41467-022-30438-w PMC9133002

[27] G. Kildisiute, M. D. Young, S. Behjati, Pitfalls of applying mouse markers to human adrenal medullary cells. Cancer Cell 39, 132–133 (2021). 33385328 10.1016/j.ccell.2020.12.006

[28] M. E. Kastriti, L. Faure, D. Von Ahsen, T. G. Bouderlique, J. Boström, T. Solovieva, C. Jackson, M. Bronner, D. Meijer, S. Hadjab, F. Lallemend, A. Erickson, M. Kaucka, V. Dyachuk, T. Perlmann, L. Lahti, J. Krivanek, J. Brunet, K. Fried, I. Adameyko, Schwann cell precursors represent a neural crest-like state with biased multipotency. EMBO J. 41, e108780 (2022). 35815410 10.15252/embj.2021108780 PMC9434083

[29] A. K. Knecht, M. Bronner-Fraser, DBHR, a gene with homology to dopamine beta-hydroxylase, is expressed in the neural crest throughout early development. Dev. Biol. 234, 365–375 (2001). 11397006 10.1006/dbio.2001.0275

[30] C. Murko, F. M. Vieceli, M. Bronner, Transcriptome dataset of trunk neural crest cells migrating along the ventral pathway of chick embryos. Data Brief 21, 2547–2553 (2018). 30761336 10.1016/j.dib.2018.11.109 PMC6288396

[31] W. Zhang, Y. Yu, F. Hertwig, J. Thierry-Mieg, W. Zhang, D. Thierry-Mieg, J. Wang, C. Furlanello, V. Devanarayan, J. Cheng, Y. Deng, B. Hero, H. Hong, M. Jia, L. Li, S. M. Lin, Y. Nikolsky, A. Oberthuer, T. Qing, Z. Su, R. Volland, C. Wang, M. D. Wang, J. Ai, D. Albanese, S. Asgharzadeh, S. Avigad, W. Bao, M. Bessarabova, M. H. Brilliant, B. Brors, M. Chierici, T.-M. Chu, J. Zhang, R. G. Grundy, M. M. He, S. Hebbring, H. L. Kaufman, S. Lababidi, L. J. Lancashire, Y. Li, X. X. Lu, H. Luo, X. Ma, B. Ning, R. Noguera, M. Peifer, J. H. Phan, F. Roels, C. Rosswog, S. Shao, J. Shen, J. Theissen, G. P. Tonini, J. Vandesompele, P.-Y. Wu, W. Xiao, J. Xu, W. Xu, J. Xuan, Y. Yang, Z. Ye, Z. Dong, K. K. Zhang, Y. Yin, C. Zhao, Y. Zheng, R. D. Wolfinger, T. Shi, L. H. Malkas, F. Berthold, J. Wang, W. Tong, L. Shi, Z. Peng, M. Fischer, Comparison of RNA-seq and microarray-based models for clinical endpoint prediction. Genome Biol. 16, 133 (2015). 26109056 10.1186/s13059-015-0694-1 PMC4506430

[32] H. Kocak, S. Ackermann, B. Hero, Y. Kahlert, A. Oberthuer, D. Juraeva, F. Roels, J. Theissen, F. Westermann, H. Deubzer, V. Ehemann, B. Brors, M. Odenthal, F. Berthold, M. Fischer, Hox-C9 activates the intrinsic pathway of apoptosis and is associated with spontaneous regression in neuroblastoma. Cell Death Dis. 4, e586 (2013). 23579273 10.1038/cddis.2013.84 PMC3668636

[33] P. Rajbhandari, G. Lopez, C. Capdevila, B. Salvatori, J. Yu, R. Rodriguez-Barrueco, D. Martinez, M. Yarmarkovich, N. Weichert-Leahey, B. J. Abraham, M. J. Alvarez, A. Iyer, J. L. Harenza, D. Oldridge, K. De Preter, J. Koster, S. Asgharzadeh, R. C. Seeger, J. S. Wei, J. Khan, J. Vandesompele, P. Mestdagh, R. Versteeg, A. T. Look, R. A. Young, A. Iavarone, A. Lasorella, J. M. Silva, J. M. Maris, A. Califano, Cross-cohort analysis identifies a TEAD4-MYCN positive feedback loop as the core regulatory element of high-risk neuroblastoma. Cancer Discov. 8, 582–599 (2018). 29510988 10.1158/2159-8290.CD-16-0861 PMC5967627

[34] T. Tao, H. Shi, M. Wang, A. R. Perez-Atayde, W. B. London, A. Gutierrez, B. Lemos, A. D. Durbin, A. T. Look, Ganglioneuromas are driven by activated AKT and can be therapeutically targeted with mTOR inhibitors. J. Exp. Med. 217, e20191871 (2020). 32728700 10.1084/jem.20191871 PMC7537400

[35] J. Barretina, G. Caponigro, N. Stransky, K. Venkatesan, A. A. Margolin, S. Kim, C. J. Wilson, J. Lehár, G. V. Kryukov, D. Sonkin, A. Reddy, M. Liu, L. Murray, M. F. Berger, J. E. Monahan, P. Morais, J. Meltzer, A. Korejwa, J. Jané-Valbuena, F. A. Mapa, J. Thibault, E. Bric-Furlong, P. Raman, A. Shipway, I. H. Engels, J. Cheng, G. K. Yu, J. Yu, P. Aspesi, M. de Silva, K. Jagtap, M. D. Jones, L. Wang, C. Hatton, E. Palescandolo, S. Gupta, S. Mahan, C. Sougnez, R. C. Onofrio, T. Liefeld, L. MacConaill, W. Winckler, M. Reich, N. Li, J. P. Mesirov, S. B. Gabriel, G. Getz, K. Ardlie, V. Chan, V. E. Myer, B. L. Weber, J. Porter, M. Warmuth, P. Finan, J. L. Harris, M. Meyerson, T. R. Golub, M. P. Morrissey, W. R. Sellers, R. Schlegel, L. A. Garraway, The cancer cell line encyclopedia enables predictive modelling of anticancer drug sensitivity. Nature 483, 603–607 (2012). 22460905 10.1038/nature11003 PMC3320027

[36] R. Cabrita, M. Lauss, A. Sanna, M. Donia, M. Skaarup Larsen, S. Mitra, I. Johansson, B. Phung, K. Harbst, J. Vallon-Christersson, A. van Schoiack, K. Lövgren, S. Warren, K. Jirström, H. Olsson, K. Pietras, C. Ingvar, K. Isaksson, D. Schadendorf, H. Schmidt, L. Bastholt, A. Carneiro, J. A. Wargo, I. M. Svane, G. Jönsson, Tertiary lymphoid structures improve immunotherapy and survival in melanoma. Nature 577, 561–565 (2020). 31942071 10.1038/s41586-019-1914-8

[37] L. Marisa, A. de Reyniès, A. Duval, J. Selves, M. P. Gaub, L. Vescovo, M.-C. Etienne-Grimaldi, R. Schiappa, D. Guenot, M. Ayadi, S. Kirzin, M. Chazal, J.-F. Fléjou, D. Benchimol, A. Berger, A. Lagarde, E. Pencreach, F. Piard, D. Elias, Y. Parc, S. Olschwang, G. Milano, P. Laurent-Puig, V. Boige, Gene expression classification of colon cancer into molecular subtypes: Characterization, validation, and prognostic value. PLOS Med. 10, e1001453 (2013). 23700391 10.1371/journal.pmed.1001453 PMC3660251

[38] S. H.-C. Cheng, T.-T. Huang, Y.-H. Cheng, T. B. K. Tan, C.-F. Horng, Y. A. Wang, N. S. Brian, L.-S. Shih, B.-L. Yu, Validation of the 18-gene classifier as a prognostic biomarker of distant metastasis in breast cancer. PLOS ONE 12, e0184372 (2017). 28886126 10.1371/journal.pone.0184372 PMC5590926

[39] M. Gautier, C. Thirant, O. Delattre, I. Janoueix-Lerosey, Plasticity in neuroblastoma cell identity defines a noradrenergic-to-mesenchymal transition (NMT). Cancer 13, 2904 (2021). 10.3390/cancers13122904 PMC8230375 34200747

[40] J. L. Harenza, M. A. Diamond, R. N. Adams, M. M. Song, H. L. Davidson, L. S. Hart, M. H. Dent, P. Fortina, C. P. Reynolds, J. M. Maris, Transcriptomic profiling of 39 commonly-used neuroblastoma cell lines. Sci. Data 4, 170033 (2017). 28350380 10.1038/sdata.2017.33 PMC5369315

[41] J. De Wyn, M. W. Zimmerman, N. Weichert-Leahey, C. Nunes, B. B. Cheung, B. J. Abraham, A. Beckers, P.-J. Volders, B. Decaesteker, D. R. Carter, A. T. Look, K. De Preter, W. Van Loocke, G. M. Marshall, A. D. Durbin, F. Speleman, K. Durinck, MEIS2 is an adrenergic core regulatory transcription factor involved in early initiation of TH-MYCN-driven neuroblastoma formation. Cancer 13, 4783 (2021). 10.3390/cancers13194783 PMC8508013 34638267

[42] T. Tao, S. B. Sondalle, H. Shi, S. Zhu, A. R. Perez-Atayde, J. Peng, S. J. Baserga, A. T. Look, The pre-rRNA processing factor DEF is rate limiting for the pathogenesis of MYCN-driven neuroblastoma. Oncogene 36, 3852–3867 (2017). 28263972 10.1038/onc.2016.527 PMC5501763

[43] S. Blockhuys, E. Celauro, C. Hildesjö, A. Feizi, O. Stål, J. C. Fierro-González, P. Wittung-Stafshede, Defining the human copper proteome and analysis of its expression variation in cancers. Metallomics 9, 112–123 (2017). 27942658 10.1039/c6mt00202a

[44] A. Furlan, V. Dyachuk, M. E. Kastriti, L. Calvo-Enrique, H. Abdo, S. Hadjab, T. Chontorotzea, N. Akkuratova, D. Usoskin, D. Kamenev, J. Petersen, K. Sunadome, F. Memic, U. Marklund, K. Fried, P. Topilko, F. Lallemend, P. V. Kharchenko, P. Ernfors, I. Adameyko, Multipotent peripheral glial cells generate neuroendocrine cells of the adrenal medulla. Science 357, eaal3753 (2017). 28684471 10.1126/science.aal3753 PMC6013038

[45] P. Shi, J. Xu, F. Xia, Y. Wang, J. Ren, P. Liang, H. Cui, MOXD1 knockdown suppresses the proliferation and tumor growth of glioblastoma cells via ER stress-inducing apoptosis. Cell Death Discov. 8, 1–9 (2022). 35393406 10.1038/s41420-022-00976-9 PMC8991257

[46] F. M. Vega, A. Colmenero-Repiso, M. A. Gómez-Muñoz, I. Rodríguez-Prieto, D. Aguilar-Morante, G. Ramírez, C. Márquez, R. Cabello, R. Pardal, CD44-high neural crest stem-like cells are associated with tumour aggressiveness and poor survival in neuroblastoma tumours. EBioMedicine 49, 82–95 (2019). 31685444 10.1016/j.ebiom.2019.10.041 PMC6945283

[47] M. C. Favrot, V. Combaret, C. Lasset, CD44–A new prognostic marker for neuroblastoma. N. Engl. J. Med. 329, 1965–1965 (1993). 10.1056/NEJM199312233292615 7504209

[48] N. Gross, K. Balmas, C. B. Brognara, Absence of functional CD44 hyaluronan receptor on human NMYC-amplified neuroblastoma cells. Cancer Res. 57, 1387–1393 (1997). 9102228

[49] M. A. Comito, V. H. Savell, M. B. Cohen, CD44 expression in neuroblastoma and related tumors. J. Pediatr. Hematol. Oncol. 19, 292–296 (1997). 9256826 10.1097/00043426-199707000-00005

[50] E. K. Siapati, E. Rouka, D. Kyriakou, G. Vassilopoulos, Neuroblastoma cells negative for CD44 possess tumor-initiating properties. Cell. Oncol. 34, 189–197 (2011). 10.1007/s13402-011-0022-z PMC13014589 21424816

[51] S. Ishida, P. Andreux, C. Poitry-Yamate, J. Auwerx, D. Hanahan, Bioavailable copper modulates oxidative phosphorylation and growth of tumors. Proc. Natl. Acad. Sci. U.S.A. 110, 19507–19512 (2013). 24218578 10.1073/pnas.1318431110 PMC3845132

[52] Y. Liao, J. Zhao, K. Bulek, F. Tang, X. Chen, G. Cai, S. Jia, P. L. Fox, E. Huang, T. T. Pizarro, M. F. Kalady, M. W. Jackson, S. Bao, G. C. Sen, G. R. Stark, C. J. Chang, X. Li, Inflammation mobilizes copper metabolism to promote colon tumorigenesis via an IL-17-STEAP4-XIAP axis. Nat. Commun. 11, 900 (2020). 32060280 10.1038/s41467-020-14698-y PMC7021685

[53] P. Lelièvre, L. Sancey, J.-L. Coll, A. Deniaud, B. Busser, The multifaceted roles of copper in cancer: A trace metal element with dysregulated metabolism, but also a target or a bullet for therapy. Cancer 12, 3594 (2020). 10.3390/cancers12123594 PMC7760327 33271772

[54] E. M. Poursani, D. Mercatelli, P. Raninga, J. L. Bell, F. Saletta, F. V. Kohane, D. P. Neumann, Y. Zheng, J. R. C. Rouaen, T. R. Jue, F. T. Michniewicz, P. Schadel, E. Kasiou, M. Tsoli, G. Cirillo, S. Waters, T. Shai-Hee, R. Cazzoli, M. Brettle, I. Slapetova, M. Kasherman, R. Whan, F. Souza-Fonseca-Guimaraes, L. Vahdat, D. Ziegler, J. G. Lock, F. M. Giorgi, K. Khanna, O. Vittorio, Copper chelation suppresses epithelial-mesenchymal transition by inhibition of canonical and non-canonical TGF-β signaling pathways in cancer. Cell Biosci. 13, 132 (2023). 37480151 10.1186/s13578-023-01083-7 PMC10362738

[55] J. Vandesompele, K. De Preter, F. Pattyn, B. Poppe, N. Van Roy, A. De Paepe, F. Speleman, Accurate normalization of real-time quantitative RT-PCR data by geometric averaging of multiple internal control genes. Genome Biol. 3, RESEARCH0034 (2002). 12184808 10.1186/gb-2002-3-7-research0034 PMC126239

[56] J. W. Bek, C. Shochat, A. De Clercq, H. De Saffel, A. Boel, J. Metz, F. Rodenburg, D. Karasik, A. Willaert, P. J. Coucke, Lrp5 mutant and crispant zebrafish faithfully model human osteoporosis, establishing the zebrafish as a platform for CRISPR-based functional screening of osteoporosis candidate genes. J. Bone Miner. Res. 36, 1749–1764 (2021). 33957005 10.1002/jbmr.4327

[57] T. Wu, E. Hu, S. Xu, M. Chen, P. Guo, Z. Dai, T. Feng, L. Zhou, W. Tang, L. Zhan, X. Fu, S. Liu, X. Bo, G. Yu, clusterProfiler 4.0: A universal enrichment tool for interpreting omics data. Innov. Camb. Mass 2, 100141 (2021). 10.1016/j.xinn.2021.100141 PMC8454663 34557778

