# Supplementary material for: MOXD1 is a lineage-specific gene and a tumor suppressor in neuroblastoma
Source: Sci Adv. 2024 Jun 21;10(25):eado1583. doi: 10.1126/sciadv.ado1583 (PMC11192077; doi:10.1126/sciadv.ado1583)
Supplement: Supplementary file 2 — Figs. S1 to S5 Tables S1 and S2 [file sciadv.ado1583_sm.pdf]

Science Advances
AAAS

Supplementary Materials for

MOXD1 is a lineage-specific gene and a tumor suppressor in neuroblastoma

Elina Fredlund *et al.*

Corresponding author: Sofie Mohlin, sofie.mohlin@med.lu.se

Sci. Adv. **10**, eado1583 (2024)
DOI: 10.1126/sciadv.ado1583

This PDF file includes:

Figs. S1 to S5
Tables S1 and S2

A. **B.**

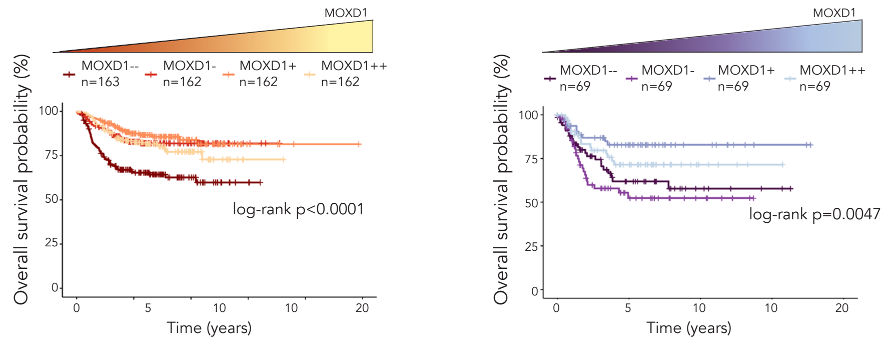

C.

	Overall survival		
	Hazard ratio	95% CI	*p*
>18mo	7.46	4.54 - 12.25	<0.001
MOXD1 --	2.19	1.48 - 3.24	<0.001
Stage 4	7.62	4.76 - 12.19	<0.001
MOXD1--	2.33	1.58 - 3.45	<0.001
MYCN ^{amp}	6.25	4.13 - 9.45	<0.001
MOXD1--	2.00	1.33 - 3.01	<0.001

D.

	Overall survival		
	Hazard ratio	95% CI	*p*
>18mo	9.27	5.99 - 14.34	<0.001
MOXD1--	1.81	1.28 - 2.54	<0.001
Stage 4	7.95	5.34 - 11.83	<0.001
MOXD1--	1.89	1.35 - 2.66	<0.001
MYCN ^{amp}	6.57	4.58 - 9.42	<0.001
MOXD1--	1.59	1.11 - 2.27	0.011

E. **F.** **G.**

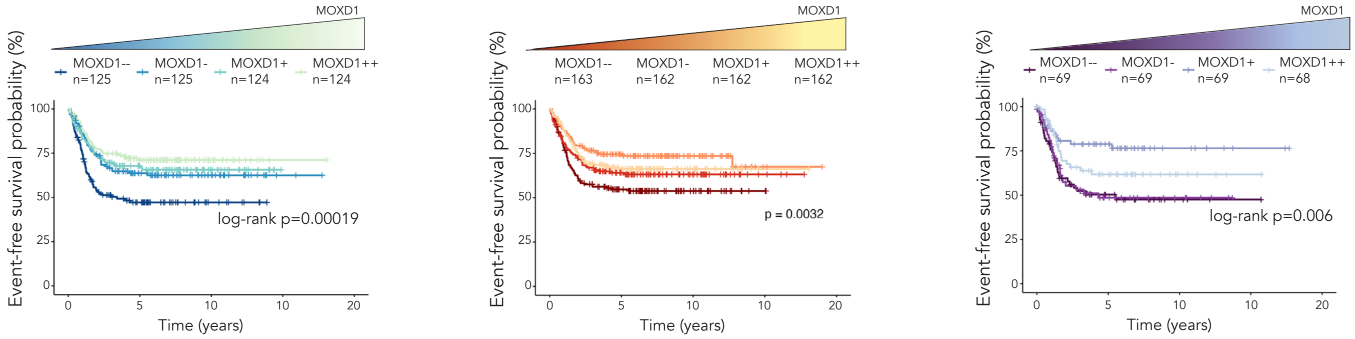

H. **I.** **J.** **K.** **L.** **M.** **N.**

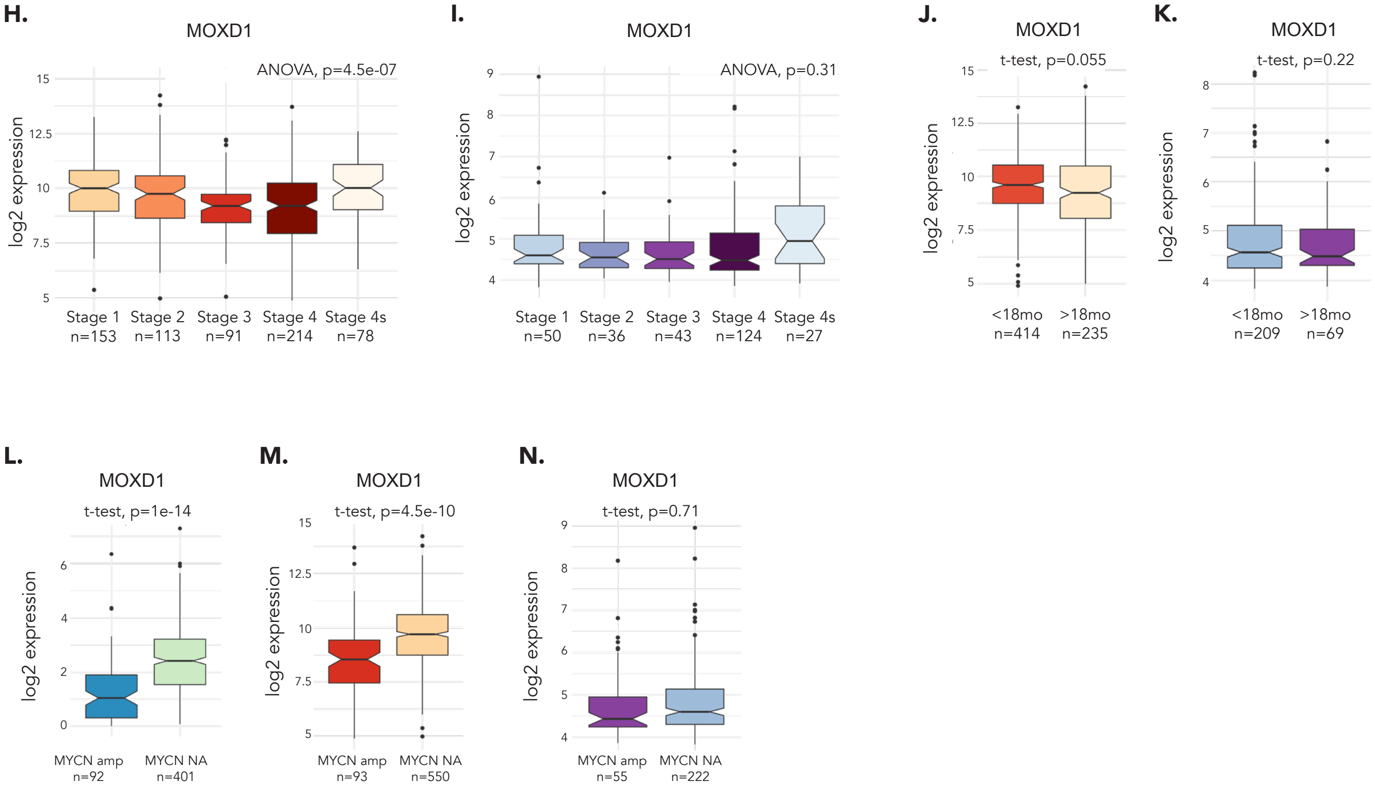

Fig. S1. Low expression of *MOXD1* correlates with poor outcome in neuroblastoma. (A–B) Kaplan-Meier overall survival curves with log-rank p-value for neuroblastoma patients in (A) the Kocak cohort (*n*=649) and (B) the NRC cohort (*n*=276). Patients are stratified into four groups (quartiles) based on *MOXD1* mRNA expression. **(C–D)** Prognostic effect of *MOXD1* mRNA expression for neuroblastoma patients in the (C) SEQC cohort and (D) Kocak cohort, comparing 1st vs 2nd, 3rd and 4th quartile of *MOXD1* expression with adjustment for age at diagnosis, INSS stage of disease and *MYCN* amplification status, respectively. Hazard ratios, 95% confidence intervals (CI) and log-rank p-values are given by multivariate Cox regression analyses. **(E-G)** Kaplan-Meier plot of event-free survival of neuroblastoma patients stratified according to quartiles of *MOXD1* expression from (E) SEQC cohort (*n*=498), (F) Kocak cohort (*n*=649), and (G) NRC cohort (*n*=276). Number of patients (*n*) is specified for each subgroup. p-value by log-rank test as indicated. **(H–I)** *MOXD1* expression across the INSS stages in neuroblastoma patients from (H) Kocak cohort and (I) NRC cohort. Number of patients (*n*) is specified for each stage and p-value by ANOVA test as indicated. **(J–K)** *MOXD1* expression in neuroblastoma patients stratified according to age at diagnosis (<18 months vs >18 months) from (J) Kocak cohort and (K) NRC cohort. Number of patients (*n*) is specified for each age group and p-value by t-test as indicated. **(L–N)** *MOXD1* expression in neuroblastoma patients stratified according to *MYCN* status; *MYCN* amplified (amp) vs *MYCN* non amplified (NA) from (L) SEQC cohort, (M) Kocak cohort, and (N) NRC cohort. Number of patients (*n*) is specified for each group and p-value by t-test as indicated.

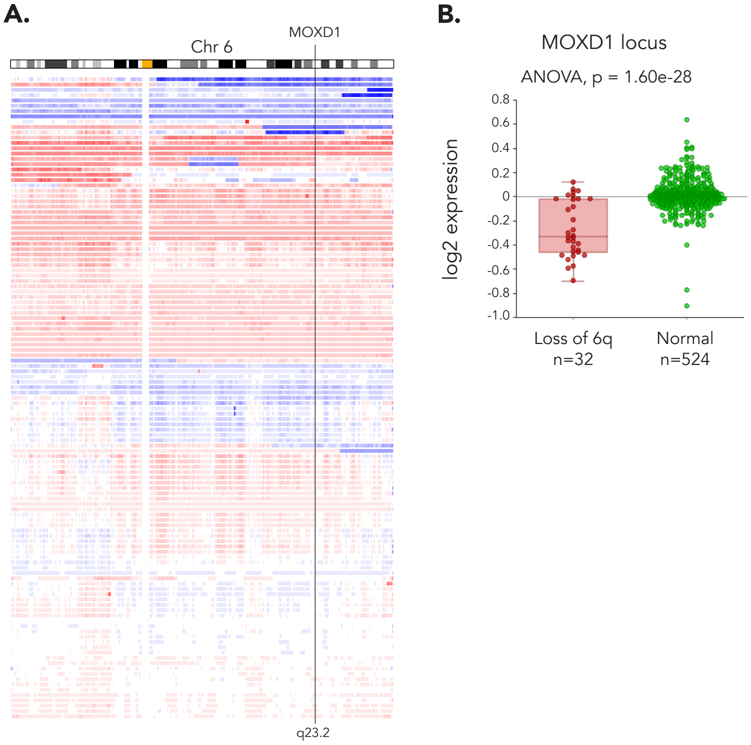
A.
MOXD1
Chr 6
q23.2
B.
MOXD1 locus
ANOVA, p = 1.60e-28
log2 expression
0.8
0.6
0.4
0.2
0
-0.2
-0.4
-0.6
-0.8
-1.0
Loss of 6q
n=32
Normal
n=524

Fig. S2. *MOXD1* locus is lost in high-risk neuroblastoma. (A) DNA copy-number aberrations on chromosome 6 from 87 neuroblastomas of all stages. The location of *MOXD1* on 6q23.2 is marked by the line. Gains, red; losses, blue. Each row represents an individual patient. **(B)** Expression of genes in the surrounding *MOXD1* locus (*ARG1, CTGF, ENPP1, ENPP3, STX7, MED23, MOXD1, OR2A4,* and *CTAGE9*) in patients with a normal 6q karyotype as compared to patients with loss of 6q (as defined in, and data from Depuydt *et al*. (*8*)). Number of patients (*n*) is specified for each group and p-value by ANOVA as indicated.

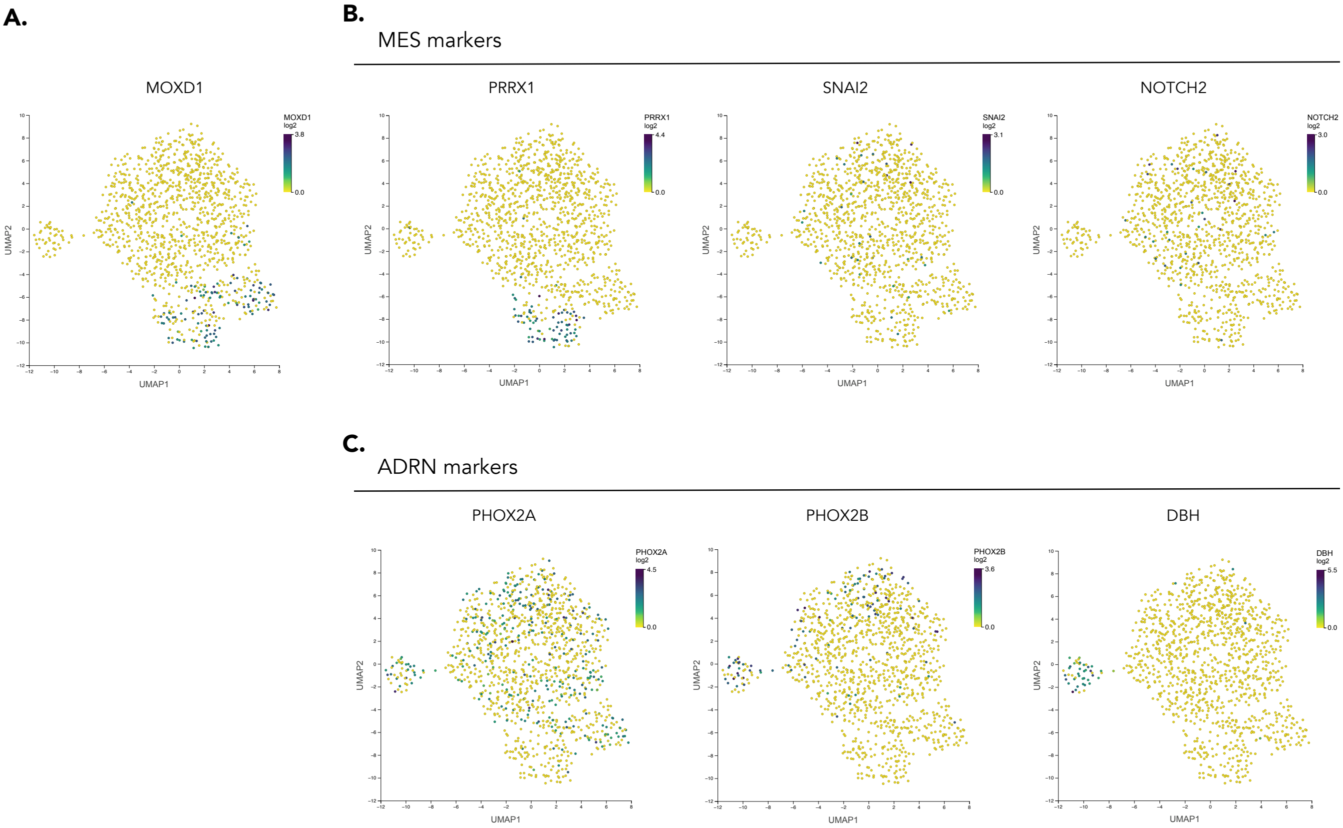
A.
B.
MES markers
C.
ADRN markers
MOXD1
PRRX1
SNAI2
NOTCH2
PHOX2A
PHOX2B
DBH
UMAP1
UMAP2

Fig. S3. SK-N-SH cells express *MOXD1* and MES- and ADRN markers. (A–C) UMAP plots from single cell analysis of the heterogeneous neuroblastoma cell line SK-N-SH (data from Gartlgruber *et al*. (*15*)). Color scale shows log2 transformed expression. **(A)** Visualization of *MOXD1* expression. **(B)** Visualization of MES markers (*PRRX1, SNAI2,* and *NOTCH2*). **(C)** Visualization of ADRN markers (*PHOX2A, PHOX2B,* and *DBH*).

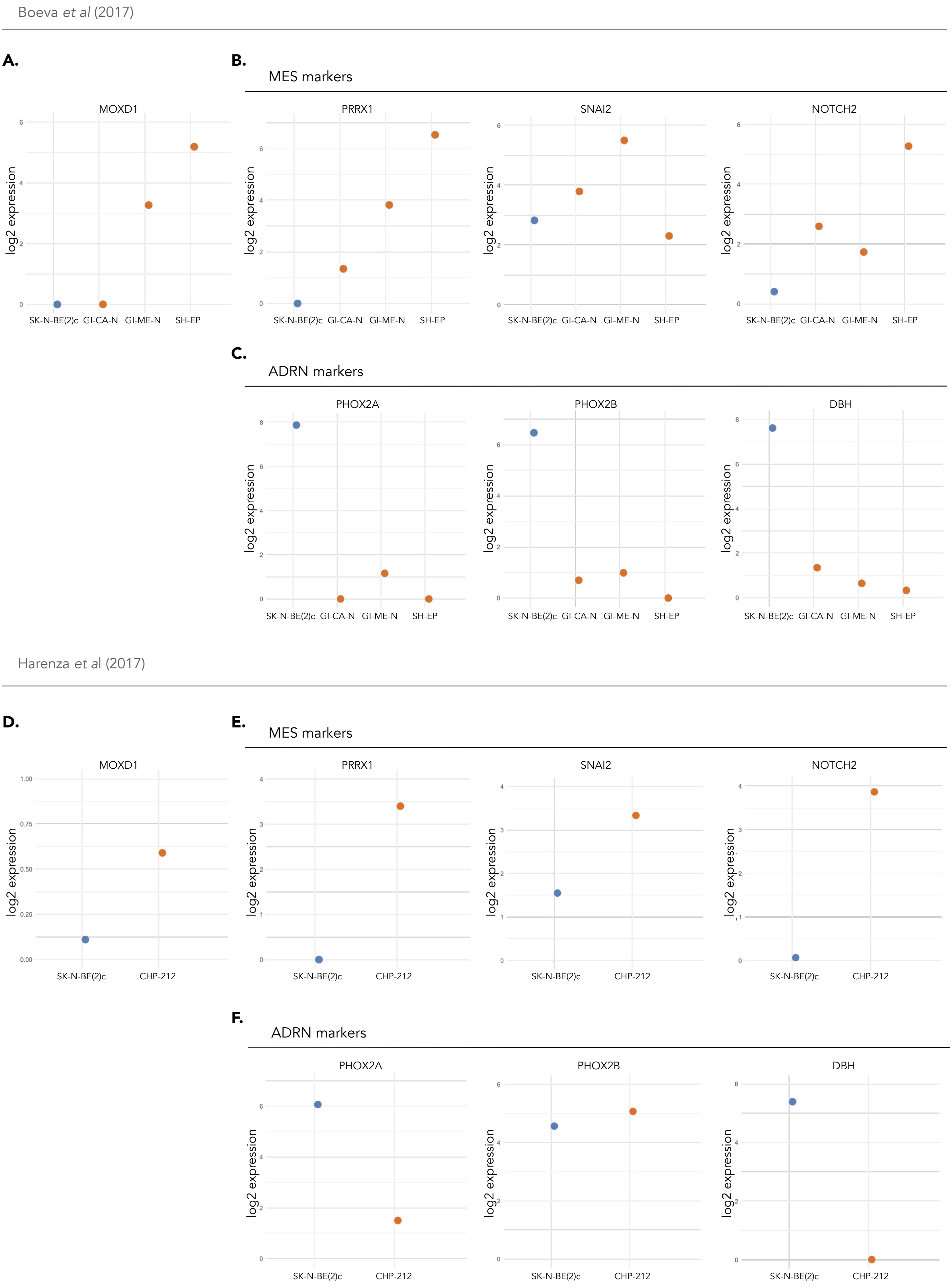
Boeva et al (2017)
A.
MOXD1
log2 expression
SK-N-BE(2)c
GI-CA-N
GI-ME-N
SH-EP
B.
MES markers
PRRX1
SNAI2
NOTCH2
C.
ADRN markers
PHOX2A
PHOX2B
DBH
Harenza et al (2017)
D.
MOXD1
CHP-212
E.
MES markers
PRRX1
SNAI2
NOTCH2
F.
ADRN markers
PHOX2A
PHOX2B
DBH

Fig. S4. *MOXD1* expression is increased in MES-like neuroblastoma cell lines. (A–C) Expression of *MOXD1* (A), MES markers (*PRRX1, SNAI2* and *NOTCH2*, (B)), and ADRN markers (*PHOX2A, PHOX2B,* and *DBH*, (C)) in SK-N-BE(2)c (ADRN-like cells) and in GI-CA-N, GI-ME-N and SH-EP (MES-like cells). RNAseq data from Boeva *et al.,* 2017 (*11*). **(D–F)** Expression of *MOXD1* (D), MES markers (*PRRX1, SNAI2* and *NOTCH2*, (E)), and ADRN markers (*PHOX2A, PHOX2B,* and *DBH*, (F)) in SK-N-BE(2)c (ADRN-like cells) and in CHP-212 (MES-like cells). RNAseq data from Harenza *et al.,* 2017 (*40*).

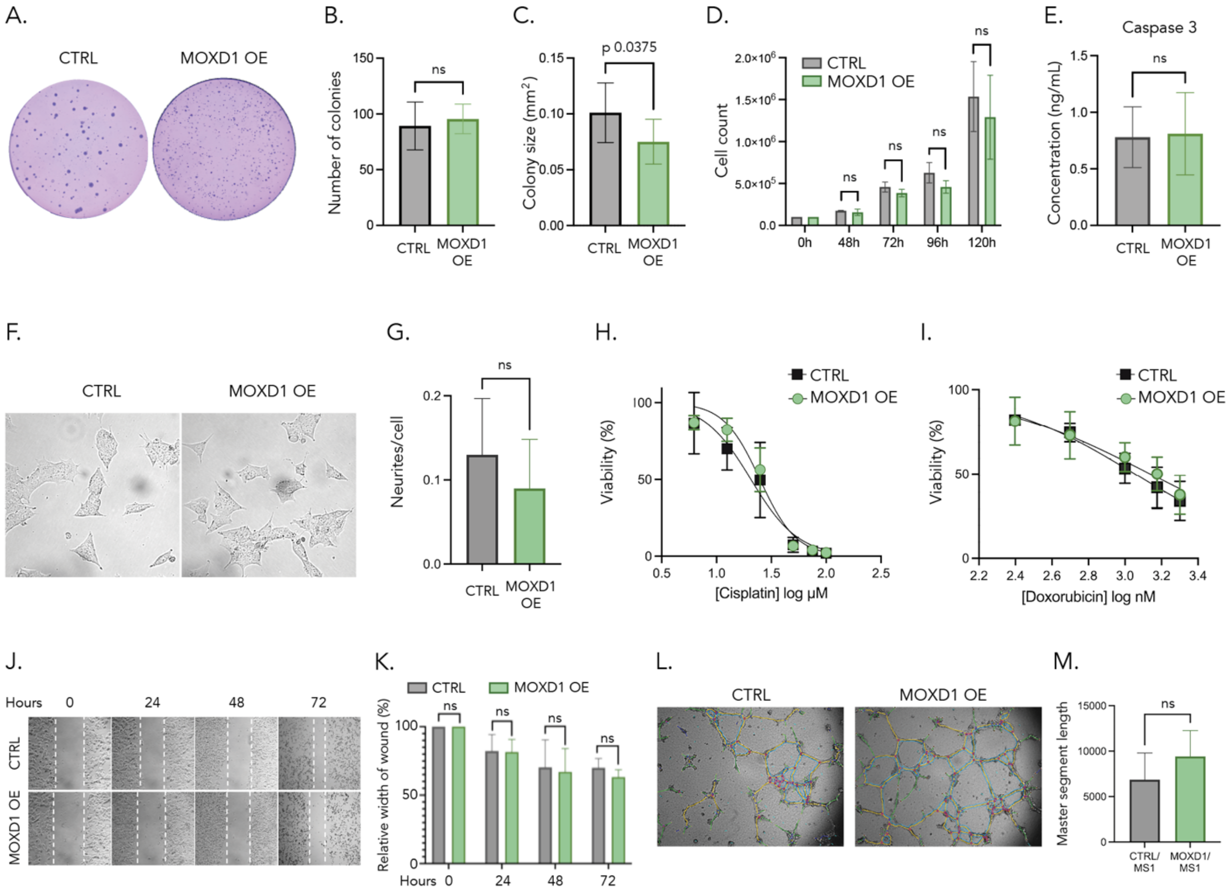
A.
CTRL
MOXD1 OE
B.
Number of colonies
ns
CTRL
MOXD1 OE
C.
Colony size (mm^2)
p 0.0375
CTRL
MOXD1 OE
D.
Cell count
CTRL
MOXD1 OE
ns
0h
48h
72h
96h
120h
E.
Caspase 3
Concentration (ng/mL)
ns
CTRL
MOXD1 OE
F.
CTRL
MOXD1 OE
G.
Neurites/cell
ns
CTRL
MOXD1 OE
H.
CTRL
MOXD1 OE
Viability (%)
[Cisplatin] log µM
I.
CTRL
MOXD1 OE
Viability (%)
[Doxorubicin] log nM
J.
Hours 0 24 48 72
CTRL
MOXD1 OE
K.
CTRL
MOXD1 OE
Relative width of wound (%)
ns
Hours 0 24 48 72
L.
CTRL
MOXD1 OE
M.
Master segment length
ns
CTRL/ MS1
MOXD1/ MS1

Fig. S5. Overexpression of *MOXD1* does not affect neuroblastoma cell differentiation or drug response *in vitro*. (A) Representative images from colony formation assay of control (CTRL) and MOXD1 overexpressing (OE) SK-N-BE(2)c cells. **(B–C)** Number (B) and size (C) of colonies formed by SK-N-BE(2)c CTRL and MOXD1 OE cells (n=3 biologically independent repeats for each group). Values are presented as mean and bars indicate standard deviation. p-value by unpaired t-test as indicated. ns, $p > 0.05$. **(D)** Proliferative capacity of SK-N-BE(2)c CTRL and MOXD1 OE cells measured by cell count at indicated time points. Values are presented as mean (n = 3 biologically independent repeats for each group) and bars indicate standard deviation. p-value by two-way ANOVA as indicated. ns, $p > 0.05$. **(E)** Detection of caspase 3 activity as determined by ELISA assay in SK-N-BE(2)c CTRL and MOXD1 OE cells. Concentrations are presented as mean (n = 3 biologically independent repeats for each group and time point) and bars indicate standard deviation. ns, $p > 0.05$. **(F)** Representative brightfield images of SK-N-BE(2)c CTRL and MOXD1 OE cells 24 hours post seeding, quantification in (G). **(G)** Blinded quantification of neurite outgrowth per cell. Values are reported as mean (n = 4 biologically independent repeats for each group), bars represent standard deviation. ns, $p > 0.05$. **(H–I)** Dose-response curves of indicated concentrations of cisplatin and doxorubicin in SK-N-BE(2) CTRL and MOXD1 OE cells. Cell viability was determined by CellTiter-Glo 48 hours post-treatment. Values are reported as mean ± SEM, (n = 3 biologically independent repeats for each group). No measured point reached $p < 0.05$. **(J)** Wound healing assay of confluent SK-N-BE(2)c CTRL and MOXD1 OE cells. Representative phase contrast images were captured at indicated time points. **(K)** Width of the wound is presented as percentage at each analyzed time point. Bars indicate standard deviation, n = 4 biologically independent repeats for each group. Statistical analysis was performed with two-way ANOVA. ns, $p > 0.05$. **(L)** Representative phase contrast images of SK-N-BE(2)c cells co-cultured with MS1 cells for 8 hours. Color overlay by ImageJ Angiogenesis Analyzer plug-in. **(M)** Images were analyzed for cellular networks using the ImageJ Angiogenesis Analyzer plug-in. Quantification of master segment length, values are presented as mean and bars indicate standard deviation (n = 2 biologically independent repeats for each group). Statistical analysis was performed with unpaired t-test. ns, $p > 0.05$.

IF antibodies				
**Primary Antibody**	**Species**	**Dilution**	**Source**	**Product #**
MOXD1 (human)	Rabbit	1:1000	Thermo Fisher	PA5-31526
MOXD1 (zebrafish)	Rabbit	1:200	Aviva	ARP62346_P0 50
**Secondary Antibody**	**Species**	**Dilution**	**Source**	
Anti-Rabbit Alexa Fluor-546	Donkey	1:1000 / 1:500	Thermo Fisher	A-10040
Anti-Rabbit Alexa Fluor-647	Donkey	1:1000	Thermo Fisher	A31573

IHC antibodies				
**Primary Antibody**	**Species**	**Dilution**	**Source**	**Product #**
MOXD1 (TMA)	Rabbit	1:1000	Thermo Fisher	PA5-31526
phospho-Histone H3 (Ser10)	Rabbit	1:200	Sigma-Aldrich	06-570
**Secondary Antibody**	**Species**	**Dilution**	**Source**	
anti-rabbit IgG (whole molecule)-Biotin antibody	Goat	1:200	Sigma-Aldrich	B-7389

Nuclear staining				
	**Species**	**Dilution**	**Source**	**Product #**
DAPI		1:3000	Dako	D3571

Table S1. List of antibodies.

Target gene	5' - 3'	
UBC (Reference gene)	Fwd	ATTTGGGTCGCGGTTCTTG
	Rev	TGCCTTGACATTCTCGATGGT
SDHA (Reference gene)	Fwd	TGGGAACAAGAGGGCATCTG
	Rev	CCACCACTGCATCAAATTCATG
YWHAZ (Reference gene)	Fwd	ACTTTTGGTACATTGTGGCTTCAA
	Rev	CCGCCAGGACAAACCAGTAT
DBH	Fwd	GCTCTCATGGAATGTCAGCTACA
	Rev	ACAGGACGCCAGCCTTGA
MOXD1	Fwd	TGAGATGTTCCAAGACAGACAA
	Rev	TGCCACACACCAAAGGTTC
NOTCH1	Fwd	CCGCAGTTGTGCTCCTGAA
	Rev	ACCTTGGCGGTCTCGTAGCT
PHOX2A	Fwd	GATGGACTACTCCTACCTCAATTCG
	Rev	GCTGCAGGCGCCAAAGT
PHOX2B	Fwd	CAGGGACCACCAGAGCAGT
	Rev	CTGCTTGCGCTTCTCGTTGA
PRRX1	Fwd	CAGAACCGAAGAGCCAAGT
	Rev	GAGTAGGATTTGAGGAGGGAAG
SNAI2	Fwd	ATGTCGGTTGTCTGGTTG
	Rev	TCTCCTGTGTTTTGTTCTTG

Table S2. List of primers for qPCR.
